# Cachexia Alters Central Nervous System Morphology and Functionality in Cancer Patients

**DOI:** 10.1002/jcsm.13742

**Published:** 2025-02-17

**Authors:** Estefania Simoes, Ricardo Uchida, Mariana P. Nucci, Fabio L. S. Duran, Joanna D. C. C. Lima, Leonardo R. Gama, Naomi A. Costa, Maria C. G. Otaduy, Fang C. Bin, Jose P. Otoch, Paulo Alcantara, Alexandre Ramos, Alessandro Laviano, Mauricio Berriel Diaz, Margaret M. Esiri, Gabriele C. DeLuca, Stephan Herzig, Geraldo Busatto Filho, Marilia Seelaender

**Affiliations:** ^1^ Cancer Metabolism Research Group (LIM26‐HCFMUSP), Department of Surgery São Paulo Brazil; ^2^ Institute for Diabetes and Cancer, Helmholtz Munich, and German Center for Diabetes Research DZD Neuherberg Germany; ^3^ Joint Heidelberg‐IDC Translational Diabetes Unit, Inner Medicine Heidelberg University Hospital Heidelberg Germany; ^4^ Mental Health Department Santa Casa de São Paulo School of Medical Sciences São Paulo Brazil; ^5^ Department of Radiology, Faculty of Medicine Laboratory of Magnetic Resonance in Neuroradiology (LIM44‐ HCFMUSP) São Paulo Brazil; ^6^ Neuroimaging Laboratory (LIM21‐HCFMUSP), institute Psychiatry University of São Paulo São Paulo Brazil; ^7^ Center for Translational Research in Oncology, Cancer Institute of the State of São Paulo University of São Paulo São Paulo Brazil; ^8^ Hospital Santa Casa de Misericórdia de São Paulo São Paulo Brazil; ^9^ Department of Clinical Surgery University Hospital USP São Paulo Brazil; ^10^ School of Arts, Sciences and Humanities University of São Paulo São Paulo Brazil; ^11^ Department of Translational and Precision Medicine Sapienza University of Rome Rome Italy; ^12^ Nuffield Department of Clinical Neurosciences University of Oxford Oxford UK; ^13^ Chair Molecular Metabolic Control Technical University Munich Munich Germany

**Keywords:** central nervous system, grey matter, human cachexia, neuroimaging, neuroinflammation, neuropathology

## Abstract

**Background:**

Cachexia is a clinically challenging multifactorial and multi‐organ syndrome, associated with poor outcome in cancer patients, and characterised by inflammation, wasting and loss of appetite. The syndrome leads to central nervous system (CNS) function dysregulation and to neuroinflammation; nevertheless, the mechanisms involved in human cachexia remain unclear.

**Methods:**

We used in vivo structural and functional magnetic resonance imaging *(Cohort 1),* as well as postmortem neuropathological analyses *(Cohort 2)* in cachectic cancer (CC) patients compared to weight stable cancer (WSC) patients. *Cohort 1* included treatment‐naïve adults diagnosed with colorectal cancer, further divided into WSC (*n* = 12; 6/6 [male/female], 61.3 ± 3.89 years) and CC (*n* = 10; 6/4, 63.0 ± 2.74 years). *Cohort 2* was composed by human postmortem cases where gastrointestinal carcinoma was the underlying cause of death (WSC *n* = 6; 3/3, 82.7 ± 3.33 years and CC *n* = 10; 5/5, 84.2 ± 2.28 years).

**Results:**

Here we demonstrate that the CNS of CC patients presents regional structural differences within the grey matter (GM). Cachectic patients presented an augmented area within the region of the orbitofrontal cortex, olfactory tract and the gyrus rectus (coordinates *X*, *Y*, *Z* = 6, 20,−24; 311 voxels; pFWE = 0.023); increased caudate and putamen volume (−10, 20, −8; 110 voxel; pFWE = 0.005); and reduced GM in superior temporal gyrus and rolandic operculum (56,0,2; 156 voxels; pFWE = 0.010). Disrupted functional connectivity was found in several regions such as the salience network, subcortical and temporal cortical areas of cachectic patients (20 decreased and 5 increased regions connectivity pattern, pFDR < 0.05). Postmortem neuropathological analyses identified abnormal neuronal morphology and density, increased microglia/macrophage burden, astrocyte profile disruption and mTOR pathway related neuroinflammation (*p* < 0.05).

**Conclusions:**

Our results indicate that cachexia compromises CNS morphology mostly causing changes in the GM of cachectic patients, leading to alterations in regional volume patterns, functional connectivity, neuronal morphology, neuroglia profile and inducing neuroinflammation, all of which may contribute to the loss of homeostasis control and to deficient information processing, as well as to the metabolic and behavioural derangements commonly observed in human cachexia. This first human mapping of CNS cachexia responses will now pave the way to mechanistically interrogate these pathways in terms of their therapeutic potential.

## 1 Introduction

Cachexia is a clinically challenging multifactorial and multi‐organ syndrome, associated with poor outcome in cancer patients, and characterised by marked involuntary weight loss, systemic inflammation, metabolic disturbances and anorexia [1, 2], ^S1^. The complexity of the symptoms renders the diagnosis difficult, thereby also compromising treatment [3], ^S2–3^. The mechanisms involved in the pathogenesis of cachexia are not fully elucidated. The current literature demonstrates that the crosstalk between the central nervous system (CNS) and the peripheral organs could play a major role in the development of the syndrome [4], ^S4^. This hypothesis is reinforced by the fact that neuroinflammation has been described in animal models of cachexia, concomitant with functional disruption of brain areas that play a key role in the development of global metabolic and behavioural control [5], ^S5^.

The CNS integrates cognitive, visual and sensory information, as well as peripheral signals (such as those conveyed by c‐reactive protein [CRP], cytokines, tumour necrosis factor [TNFα], interleukin‐1 [IL1], neuropeptides and hormones) with impact on neural circuits controlling feeding behaviour, energy homeostasis and neurogenesis [6, 7], ^S6–7^. The inflammatory markers sensed by the CNS constantly activate and sensitise the microglia, as well as CNS‐infiltrating immune cells mediating neuroinflammatory responses [8], ^S8^. In the face of systemic inflammation, microglia activation triggers and perpetuates neuroinflammation, potentially contributing to many CNS disorders [9], ^S9–10^. In animal models, the intrahypothalamic concentration of TNFα and IL1 is increased as a consequence of the systemic inflammation that characterises cachexia [4]. Norden et al. (2016) have shown that the sequential activation of microglia and astrocytes leads to glial profile transformation, with cells becoming neurotoxic, displaying phagocytic potential and the capacity to release proinflammatory mediators, different from those implicated in the systemic scenario [10]. In the orchestration of a response to the presence of inflammatory stimuli, mTOR pathway‐dependent microglia priming and astrogliosis occur [11], ^S11^. Under this perspective, the mechanistic target of rapamycin (mTOR) pathway emerges as a possible crucial mediator of both innate and adaptive immune cells responses [12]. Given the crucial role of the mTOR pathway in regulating anabolic processes, brain control of food intake, synaptic plasticity could be relevant in cancer cachexia pathophysiology [13]. Dysregulation of mTOR signalling is linked to various pathologic states, with particularly detrimental outcomes for nervous system development and function [14], ^S12^. mTOR pathway hyperactivation and concomitant neuroinflammation can cause severe negative impact on CNS function leading to synaptic dysfunction, neurogenesis inhibition, microglia priming, neural death, cognitive decline, loss of memory, behavioural changes and brain damage [15], ^S13^. mTOR is composed by two complexes under different regulatory control (mTORC1 and mTORC2) [16]. In animal studies, disruptions in several regulators of mTOR (upstream TSC1 and TSC2, as well as downstream target p70S6K) affect immune responses, metabolism, proliferation, cell survival and growth [17], ^S14^.

**FIGURE 1:**
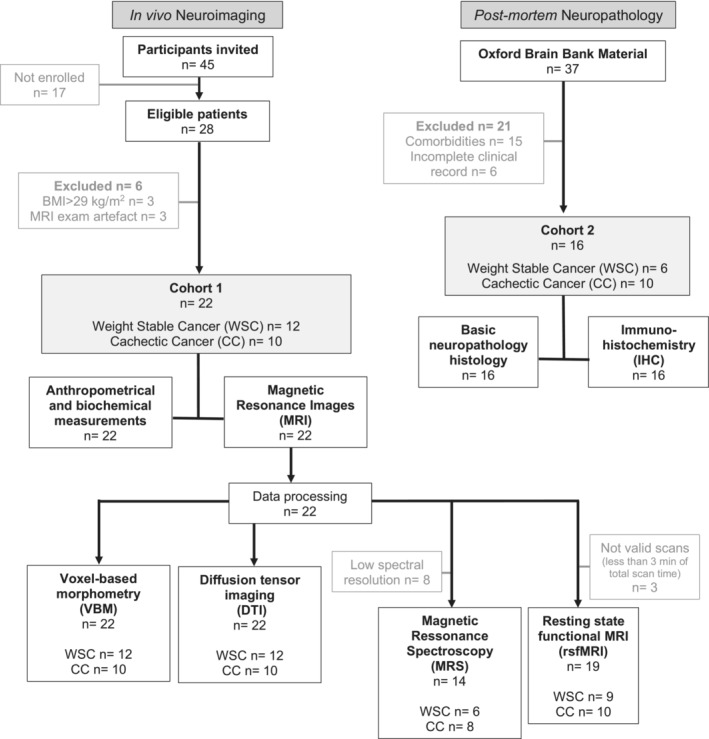
Flowchart for the in vivo neuroimaging study in patients and for the postmortem neuropathological analysis in cadaver samples.

The aim of the present study was to examine CNS morphology and function, neuronal morphology and CNS‐immune cell profile in cachectic cancer (CC) patients, compared to weight stable cancer (WSC) patients, using in vivo structural and functional magnetic resonance imaging (MRI), as well as postmortem neuropathological analyses. Nevertheless, this is, to the best of our knowledge, the first study to provide the characterisation of the human brain in cancer cachexia research, as well as to combine in vivo neuroimaging and postmortem brain tissue analyses (see flowchart Figure 1). Our study suggests that cachexia alters CNS grey matter (GM) morphology, functional connectivity (FC), neuronal morphology, neuroglia profile and induces neuroinflammation.

## 2 Methods

### 2.1 Study Description

This is a case–control study to evaluate the CNS of CC patients. As showed in the flowchart (Figure 1), the data presented in this manuscript were obtained from an in vivo neuroimaging study where patients were recruited in the University Hospital and Santa Casa Hospital in São Paulo (Brazil) and a postmortem neuropathological study where brain tissue was obtained from the Oxford Brain Bank (England).

## 3 In Vivo Neuroimaging

Ethical approval for this study was granted by the University of Sao Paulo Biomedical Sciences Institute Ethics Committee (CAAE: 54931116.6.0000.5467), the University Hospital Ethics Committee (CAAE: 54931116.6.3001.0076) and the Santa Casa Hospital Ethics Committee (CAAE: 54931116.6.3002.5479) in accordance with the Declaration of Helsinki (2013). All participants signed an informed consent form prior to entering the study.

### 3.1 Cohort 1—São Paulo, Brazil

Eligible patients were treatment‐naïve adults, 40–80 years old diagnosed with colorectal cancer. Cancer patients were further divided into WSC (*n* = 12) and CC (*n* = 10), according to the criteria proposed by Evans et al. [1]. Cachectic patients should present at least 5% of involuntary weight loss in the previous 6 months or BMI < 20 kg/m^2^ and three out of five of the following criteria: (1) decreased muscle strength, (2) fatigue, (3) anorexia, (4) low fat‐free mass index or (5) abnormal biochemistry characterised by increased inflammatory biomarkers (PCR > 5 mg/L; IL‐6 > 4 pg/mL), anaemia (Hb < 12 g/dL) or hypoalbuminemia (< 3.2 g/dL).

The inclusion criteria were as follows: not having received anticancer or continuous anti‐inflammatory treatment, willingness to participate and body mass index (BMI) < 29 kg/m2. The exclusion criteria were as follows: liver failure, renal failure, AIDS, inflammatory diseases of the bowel and autoimmune disorders. Cohort 1 general patient characteristics are summarised in Table 1.

**TABLE 1:** General characteristics of Cohort 1.

Characteristics	WSC	CC	*p‐value*
*N*	12	10	
Male/female (*n*)	6/6	6/4	0.639
Age (years)	61.3 ± 3.89	63.0 ± 2.74	0.740
Height (m)	1.59 ± 0.02	1.61 ± 0.03	0.727
Previous BM (kg)	66.1 ± 4.09	61.8 ± 2.99	0.430
Current BMI (kg/m^2^)	24.9 ± 1.15	21.2 ± 0.90	**0.026**
Δ Weight (%)	2.14 ± 1.28	12.8 ± 2.27	**0.0003**
Initial tumour stage (I and II)	2	4	0.232
Final tumour stage (III and IV)	7	4	
Haemoglobin (g/dL)	13.4 ± 0.56	12.1 ± 0.85	0.224
CRP (mg/L)	1.44 ± 0.52	5.73 ± 1.93	**0.038**
Albumin (g/dL)	4.06 ± 0.23	3.74 ± 0.21	0.872
IL1 (pg/mL)	1.23 ± 0.24	1.37 ± 0.76	0.224
IL6 (pg/mL)	2.29 ± 0.48	5.11 ± 1.62	0.099
IL8 (pg/mL)	11.7 ± 2.07	33.3 ± 8.77	**0.048**
IL10 (pg/mL)	3.21 ± 0.57	4.53 ± 0.65	0.179

*Note:* Data are presented as mean ± SEM. Significant differences between the groups were tested employing the unpaired *T*‐test. Chi‐squared test was employed for age and tumour staging. Significant *p*‐values in bold.

Abbreviations: BM, body mass; BMI, body mass index; CC, cancer cachexia; CRP, c‐reactive protein; ; IL, interleukin; *N*, sample size; WSC, weight stable cancer.

### 3.2 Blood Collection

Approximately 20 mL of fasting blood was collected by a trained health professional. Blood was placed in BD Vacutainer® Tubes without anticoagulant, centrifuged to obtain the serum, transferred to plastic microtubes and stored in a freezer at −80°C.

### 3.3 Clinical and Biochemical Parameters Assessment

Biochemical parameters were quantified in the automatic analyser Labmax 240 (Labtest®), employing commercial kits for CRP and Albumin (Cat#331 and #19 Diagnostic Labtest, respectively). Haemoglobin content was obtained from hospital records.

### 3.4 Analysis of Protein Content

Serum samples were analysed by Luminex®xMAP™ technology and performed according to the manufacture's protocol: Human Cytokine/Chemokine Magnetic Bead Panel Cat. #HCYTMAG‐60 K‐PX30. Serum samples were incubated with the mixture of Multiplex Magplex microspheres, covered with the specific antibodies, incubated with a mixture of biotinylated capture antibodies and streptavidin labelled with phycoerithrin. The microspheres were analysed with the phycoerithrin Magpix® instrument (Life Technologies). Protein quantification was preceded with the equipment‐specific software (xPONENT® 4.2), and the obtained data were analysed in the MILLIPLEX® Analyst 5.1 Software.

### 3.5 MRI

#### 3.5.1 MRI Image Acquisition

MRI scanning was performed on a 3 T Philips® Achieva MR system with 32‐channel head coil at the Institute of Radiology of the Faculty of Medicine, University of São Paulo. Patients were in a fasted state during the exam. Four major MRI techniques were acquired and analysed: voxel‐based morphometry (VBM), diffusion tensor imaging (DTI), magnetic resonance spectroscopy (MRS) and resting state functional MRI (rsfMRI). Detailed MRI acquisition description is presented in the Supporting Information Methods S1.

#### 3.5.2 VBM Data Processing

All images were reviewed by a neuroradiologist with the purpose of identifying artefacts during image acquisition and the presence of silent gross brain alterations. VBM processing was performed using the Statistical Parametric Mapping (SPM) programme, version 12 (Wellcome Trust Centre of Neuroimaging, London, United Kingdom, http://www.fil.ion.ucl.ac.uk/spm) and implemented in MATLAB R2012a (MathWorks, Sherborn, Massachusetts). VBM data processing is extensively described in the Supporting Information Methods S2.

#### 3.5.3 DTI Data Processing

The diffusion tensor images were analysed using FMRIB Software Library (FSL) version 5.0.6 (http://fsl.fmrib.ox.ac.uk/fsl/fslwiki/FSL) ^S15^. DTI data processing extensively described in the Supporting Information Methods S3.

#### 3.5.4 rsfMRI Data Processing

rsfMRI data were preprocessed following the conservative settings pipeline of CONN toolbox v18c ^S16^ in conjunction with SPM 12 (release 7487). SPM 12 was implemented in MATLAB (version R2015a) to identify functional outlier scans based on the artefact detection toolbox (ART) thresholds, with the global mean signal intensity that exceeded 3 standard deviations or slice‐to‐slice movement that exceeded 0.5 mm. rsfMRI data processing is extensively described in the Supporting Information Methods S4.

#### 3.5.5 MRS Quantification

MRS analyses were performed using the LCModel software (version 6.3) to quantification metabolite levels of glutamate (Glu), sum of glutamate and glutamine (Glx), N‐acetylaspartate (NAA), myoinositol (mI), sum of glycerophosphocholine (GPC) and phosphocholine (PCh) and creatine (Cr) ^S17^. MRS quantification is extensively described in the Supporting Information Methods S5.

## 4 Postmortem Neuropathological Analysis

Human postmortem brain tissue was obtained from the Oxford Brain Bank having been collected in accordance with the UK Human Tissue Act, with informed consent provided for all donations (Oxford Brain Bank Research Ethics Committee approval number 15/SC/0639, TW536).

### 4.1 Cohort 2—Oxford, United Kingdom

Eligible patients were adults, 40–80 years old, diagnosed with gastrointestinal carcinoma (GIC). A human autopsy cohort of cancer patients was created and divided into two cohorts: WSC (*n* = 6) and CC (*n* = 10), following the criteria proposed by Evans et al. [1] and according to medical records and clinical summary data. Cohort 2 general patient characteristics are summarised in Table 2, and the Supporting Information Table S1 demonstrates patient demographics. Evidence of cognitive impairment was assessed by CERAD‐AD‐related pathology and Braak staging consisting in evaluation of neurofibrillary tangles (NFT's).

**TABLE 2:** Case demographics descriptive statistics of Cohort 2.

Characteristics	WSC	CC	*p‐value*
*N*	6	10	
Male/female (*n*)	3: 3	5: 5	> 0.999
Age (years)	82.7 ± 3.33	84.2 ± 2.28	0.701
Tumour stage: (nonmetastatic: metastatic)	2: 4	3: 7	0.890
PM interval (hours)	44.3 ± 10.9	49.1 ± 8.64	0.738

*Note:* Data are presented as mean ± SEM. Significant differences between the groups were tested using unpaired *T‐*test. Chi‐squared test was employed for sex and age.

Abbreviations: CC, cancer cachexia; *N*, sample size; PM, postmortem; WSC, weight stable cancer.

### 4.2 Tissue Preparation and Sampling

Whole postmortem brains were fixed by immersion in 10% formalin solution for ≥ 4 weeks and ≤ 4 months. Cases with longer fixation times were omitted to ensure the preservation of antigenicity for immunohistochemistry (IHC) ^S18^. Total and notional formalin‐fixed grey and white matter (WM) cross‐sectional areas were obtained for each area of interest (hypothalamus, amygdala, caudate nucleus and putamen). Staining was performed on sections of 6‐μm thickness (Leica RM2135) and mounted on glass slides (Superfrost plus slides 25 × 75 × 1mm, Thermo Scientific™ J1800AMNZ).

### 4.3 Basic Neuropathology Histology

To analyse neuronal density and neuronal counts, all cases were stained with haematoxylin and eosin (H&E, using Thermo Shandon Linistain™ Random Access Stainer). Slides were deparaffinised and rehydrated using a standard rehydration curve (3 × 5 min Xylene, 2 × 100% Ethanol, 70% Ethanol, H_2_0). The following steps were performed for H&E staining: (1) 3x Harris's haematoxylin (Thermo Scientific™ 6 765 003) and H_2_0; (2) 0.4% acid alcohol and H_2_0; (3) Scot's tap water (Thermo Scientific™ 6 769 001) and H_2_0; (4) 2x 0.25% aqueous eosin (Thermo Scientific™ 6 766 009) + 0.025% acetic acid and H_2_0; (5) dry drain and dehydrate the slides (H_2_O, 70% Ethanol, 2x100% Ethanol, 3x Xylene); and (6) mounting the slides using DPX (Fisher Scientific, 12 658 646).

### 4.4 Immunohistochemistry (IHC)

Caudate nucleus, putamen, amygdala and hypothalamus from all cases were immunolabelled using immune cells (microglia/macrophages) markers like Iba1 (Wako 019‐19 741), GFAP (Dako z0334) and CD68 (Dako M0876). Also, mTOR‐related pathway proteins were assessed: TSC1 (Abcam ab40872), TSC2 (Abcam ab32554), mTOR (Cell signalling 2983), pmTOR (Cell signalling 2976), p70S6K (Cell signalling 2708) and p‐p70S6K (Invitrogen PA5‐38307markers). Detailed IHC protocol is presented in the Supporting Information Methods S6.

### 4.5 Image Acquisition

H&E slides were digitally captured using an Olympus BX43 microscope (Olympus corporation) and an Axiocam MRc Zeiss Cam (Carl Zeiss Light Microscopy) under a 400x magnification.

Whole IHC slide images were digitally captured by an Aperio Scanscope® AT slide scanner (Leica Microsystems, Wetzlar, Germany; images viewable at x1 – x400 magnification). Specific cell and pixel counting algorithms for each marker (Supporting Information Table S3) were used to quantify the density of immunopositive pixels (per μm^2^ in each selected brain region (Figure 4c) using Qupath v.0.1.2, a software for digital pathology image analysis ^S19^.

### 4.6 Regional Analysis

After staining, slides were blinded and counted. All analysed regions (caudate nucleus, putamen, amygdala and hypothalamus) were quality controlled for tissue integrity and artefacts. Cases were omitted from the analysis when any of these features obscured the analysis by being located in the regions of interest (ROIs). A minimum of three samples were counted per analysed group. Quantitative and semiquantitative analyses were performed; see detailed description in the supplemental information (the Supporting Information Methods S7).

## 5 Statistical Analysis and Correlation Analysis

Data are expressed as mean ± SEM. Preliminary analyses were carried out to ensure that the assumptions of normality or homoscedasticity were not violated. The Chi‐square test was used for dichotomised values. Unpaired *T*‐test was employed for parametric data. Significance was considered at *p*‐value < 0.05. Statistical analyses were performed using Graphpad Prism 8.0. Detailed description of the neuroimaging statistical analysis is presented in the Supporting Information Methods S8.

### 5.1 Correlation Analysis

Nonadjusted Spearman correlations between anthropometrical and biochemical measurements and cytokines levels were performed for CC and WSC patients. Pairwise computation of the correlation was carried out excluding the patients lacking data for a specific variable using standard GNU R functions. The R code used to calculate the correlations and generate the graphs is available online at https://github.com/amphybio/Simoes2024. The *p*‐values indicating the statistical significance are given in red or in black, respectively, at the bottom of each dispersion plot when *p* < 0.05 or *p* ≥ 0.05.

## 6 Results

For the in vivo neuroimaging study, 22 colorectal cancer patients (WSC, *n* = 12 and CC, *n* = 10) were studied. The general characteristics of Cohort 1 are listed in Table 1. There were no differences in the distributions of gender, age, height or tumour staging between groups. Body mass (BM) 6 months prior to the start of the study (as reported by the patients) showed no difference (*p* = 0.430), whereas current body weight at time of analysis was lower for CC (*p* = 0.026). Cachectic patients showed marked weight loss (−12.8%, *p* = 0.0003) and increased content of circulating markers of cachexia [such as CRP, *p* = 0.038)] relative to WSC, in line with Evans et al. 2008 [1]. Also, IL8 content was increased in CC (*p* = 0.048).

### 6.1 CNS Morphometry and Connectivity Are Altered in Cachectic Patients

#### 6.1.1 GM of Appetite‐Behaviour‐Related Regions Is Affected in the Cachectic patient's Brain

**FIGURE 2:**
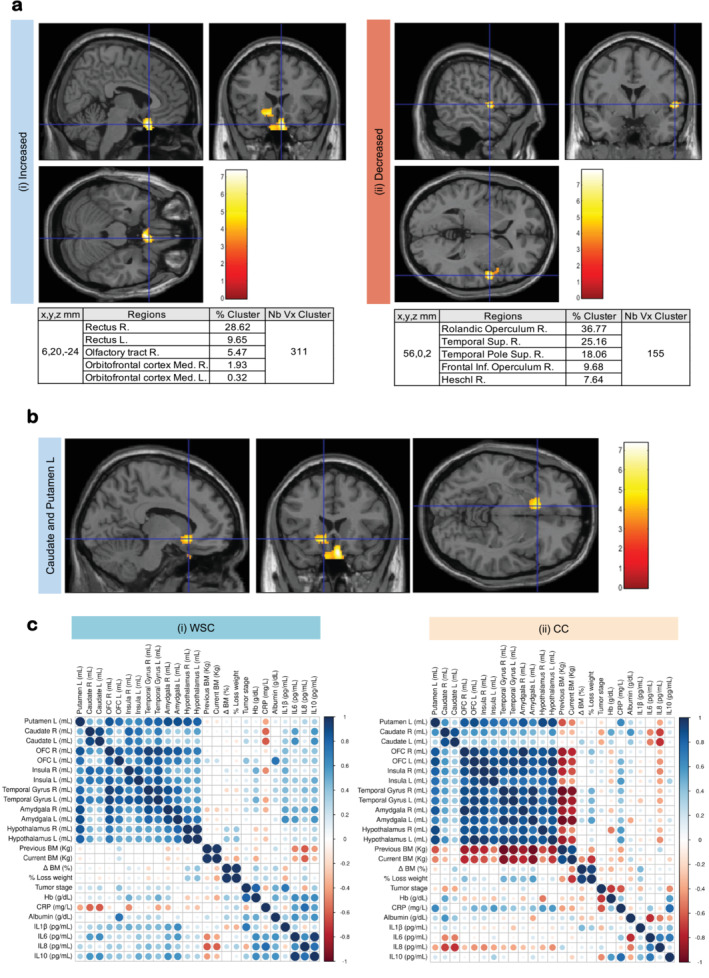
Morphometric alterations in human cachectic brain detected by VBM analyses. (a) (i) Increased grey matter regions in CC; (ii) decreased grey matter regions in CC. Coloured bar represents the *T*‐value (post‐hoc tests) for the voxel of maximum statistical significance within each cluster. *x, y, z*: coordinates; Nb, number; Vx, voxel; R, right; L, left; Med, medial; Sup, superior; Inf, inferior. (b) Increased ROIs in CC patients; (c) nonparametric Spearman correlation analysis between biological variables and ROIs: (i) correlations in WSC patients and (ii) correlations in CC patients. Each scatterplot on the panel corresponds to the pair of variables indicated on the labels on the horizontal and vertical axes. The value of the Spearman coefficient (*ρ*) is indicated by colour according to the colour scale on the right. BM, body mass; CC, cancer cachexia, *n* = 10; CRP, c‐reactive protein; IL: interleukin; L, left; ORF, orbitofrontal cortex; R, right; WSC, weight stable cancer; *n* = 12.

VBM analyses showed that the absolute total volumes of GM, WM, cerebrospinal fluid (CSF), total brain volume (TBV; calculated as GM + WM) and total intracranial volume (TIV; calculated as GM + WM + CSF) did not differ between WSC and CC (Extended Data S1). Nevertheless, the whole‐brain exploratory between‐group comparisons revealed that a GM region of 311 voxels was increased in CC patients, as compared with WSC (pFWE = 0.023, Figure 2a (i), Extended Data S2). The augmented region corresponded to the orbitofrontal cortex, olfactory tract and the gyrus rectus (coordinates *X, Y, Z* = 6, 20. −24). Furthermore, CC presented a reduced GM region of 156 voxels (pFWE = 0.010; coordinates 56, 0, 2; Figure 2a (ii); Extended Data S2) encompassing structures such as the superior temporal gyrus and rolandic operculum. No significant differences were found in whole‐brain exploratory analyses for the WM (pFWE > 0.05; Extended Data S3).

Following the study of total volumes, we performed VBM analyses of several ROIs though small volume correction (SVC) method. In the GM, SVC‐based analyses were guided by a priori selection of brain regions (amygdala, caudate, putamen and hypothalamus). Those ROIs were selected considering previous studies in healthy individuals [18], ^S20^, obese patients [19], ^S21^ and adults with eating disorders [20], ^S22^. Amygdala, caudate, putamen and hypothalamus were postulated as important brain regions which are involved in eating behaviour, ingestion, regulation of homeostasis and behaviour, among other [21], ^S23^. In the present study, SVC analysis showed an increased volume of the left caudate and putamen region (coordinates −10, 20, −8, 110 voxel), in CC as compared with WSC (pFWE = 0.005; Figure 2b). No differences were found in the amygdala or hypothalamus (pFWE > 0.05). SVC‐based analyses were also performed in a priori selection of WM regions: internal and external capsule, cingulum, fornix/stria terminalis and corpus callosum. These WM regions were chosen as they surround and are highly connected to our GM ROIs. No significant differences were found in the SVC‐based analyses in the WM (pFWE > 0.05). Moreover, DTI analysis did not show differences in fractional anisotropy between CC and WSC (Extended Data S4).

Correlation analysis between ROIs volume and clinical data was carried out to investigate how the morphological changes between groups (increase and decrease in the GM of CC patients) could bear a parallel with the changes in any given set of anthropometric data, biochemical factors or with the content of cytokines in the serum (Figure 2c). For WSC, solely, there was a positive correlation between albumin and orbitofrontal cortex L volume (*r* = 0.733; *p* = 0.021) (Figure 2c (i)). Furthermore, CC presented negative correlations between current BM and the following ROI volumes: amygdala L (Left) (*r* = −0.928; *p* = 0.0003), amygdala R (Right) (*r* = −0.783; *p* = 0.017), orbitofrontal cortex L (*r* = −0.695; *p* = 0.038), orbitofrontal cortex R (*r* = −0.762; *p* = 0.017), temporal gyrus L (*r* = −0.837; *p* = 0.005), temporal gyrus R (*r* = −0.921; *p* = 0.0004), frontal gyrus (*r* = −0.821; *p* = 0.007) and hypothalamus L (*r* = −0.711; *p* = 0.032). The content of IL8 was negatively correlated to the left (*r* = −0.733; *p* = 0.031) and right (*r* = −0.683; *p* = 0.050) caudate nucleus volumes in cachectic patients (Figure 2c (ii)). Overall, these findings indicated that cachectic patients weight loss is negatively correlated to the volume of several brain regions involved in food intake, appetite, olfaction, taste and emotion.

#### 6.1.2 rsfMRI Shows Disrupted FC in Cachectic Patients

**FIGURE 3:**
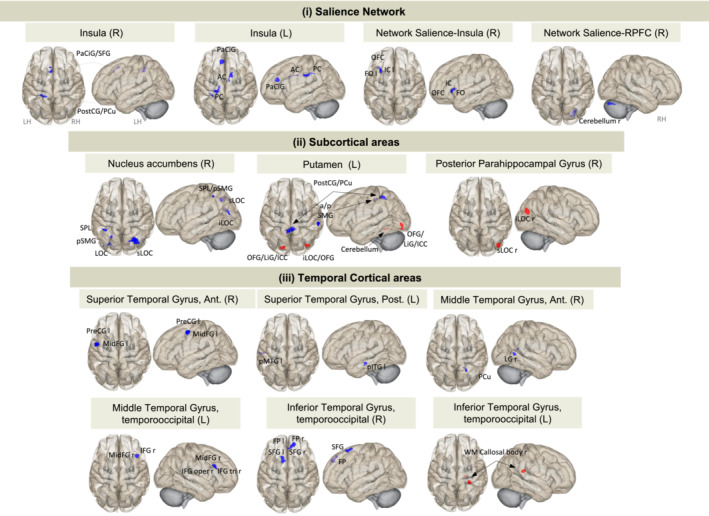
Disrupted functional connectivity in cachectic human brain assessed with rsfMRI. The FC maps display the parametric statistics (Gaussian random field theory) cluster threshold: *p* < 0.05 cluster‐size p‐FDR corrected; voxel threshold: *p* < 0.001 p‐uncorrected. AC, anterior cingulate gyrus; Ant. or a, anterior; FG, frontal gyrus; FG oper, frontal gyrus pars opercularis; FG tri, frontal gyrus pars triangularis; FO, frontal operculum; FP, frontal pole; i, inferior; IC, insular cortex; left; ICC, intracalcarine cortex; ITG, inferior temporal gyrus; ; LG, lateral gyrus; L or l, left; LH, hemisphere; LOC, lateral occipital cortex; Mid, middle; LiG, lingual gyrus; MTG, middle temporal gyrus; OFC, orbitofrontal cortex; OFG, occipital fusiform gyrus; PaCiG, paracingulate gyrus; PC, posterior cingulate gyrus; PCu, precuneous; PreCG, precentral gyrus; Post or p, posterior.; PostCG, postcentral gyrus; R or r, right; RH, right hemisphere; RPFC, right prefrontal cortex; s, superior; SFG, superior frontal gyrus; SMG, supramarginal gyrus; SPL, superior parietal lobule; WM, white matter.

Our rsfMRI study was performed adopting seed‐to‐voxel analyses from a priori ROI, such as the salience network, subcortical and temporal areas (13 seeds, as shown in Figure 3). We prioritised studying the interactions between networks or brain regions involved in food reward, body weight regulation, emotional and sensory stimuli [22], ^S24–26^.

Differences in resting‐state FC were found among WSC and CC. The results reveal a predominant and significant pattern of decreased connectivity (20 negative connections, Figure 3 in blue) rather than increased connectivity (5 positive connections, Figure 3 in red) in CC patients compared to WSC, as illustrated in Figure 3 (pFDR < 0.05, statistical values are listed in Extended Data S5).

Several regions of the salience network exhibited exclusively negative connectivity in CC patients relative to WSC patients (Figure 3(i), Extended Data S5). On the one hand, the bilateral insular cortex seeds are negatively interacting with medial and anterior brain regions, such as the anterior and posterior cingulate cortex (AC and PC), paracingulate cortex/superior frontal gyrus (PaCiG), and the left orbital frontal cortex (OFC)/frontal opercular (FO). On the other hand, the rostral prefrontal cortex (RPFC) seed connected with the cerebellum.

Considering the subcortical seeds, significant differences in FC (positive and negative) were observed between subcortical areas and posterior brain regions (Figure 3(ii), Extended Data S5). Decreased FC was found between the right nucleus accumbens seed and the bilateral lateral occipital cortex (LOC) and the left superior parietal lobule (SPL)/posterior supramarginal gyrus (SMG). The left putamen seed had negative FC with the SMG, PC and Precuneous (PCu) and also showed positive FC with bilateral occipital fusiform gyrus (OFG)/LOC/lingual gyrus (LiG). OFG/LOC/LiG cluster had also an increased FC to the right posterior parahippocampal gyrus seed.

The superior and middle temporal gyrus (STG and MTG) seeds had decreased and increased FC in the CC patients (Figure 3(iii), Extended Data S5). CC patients had negative FC in the right anterior STG seed with the left middle frontal gyrus (MidFG)/precentral gyrus (PreCG), the posterior STG with posterior medial/inferior TG (pMTG/pITG), the anterior MTG with lateral gyrus (LG) and PCu, the left temporooccipital MTG with the right frontal areas (middle, inferior frontal gyrus pars triangularis and opercularis) and the right inferior temporal gyrus (ITG) with a bilaterally frontal pole and superior frontal gyrus. In contrast, the left ITG showed increased FC with the right callosal body of CC compared to WSC patients.

Finally, we also performed proton magnetic resonance spectroscopy (1H‐MRS) to examine the in vivo hypothalamic secretion of several metabolites (such as glutamate, N‐acetylaspartate). No differences were found between CC and WSC patients (Extended Data S6).

### 6.2 Postmortem Cachectic Human Brain Tissue Features Morphological and Neuroglia Alterations

To confirm the alterations found using in vivo neuroimaging, we directly studied postmortem brain samples from the ROIs a priori selected in our SVC‐based analyses. Samples from 16 deceased gastrointestinal carcinoma cancer patients (6 WSC, and 10 CC) were included for the postmortem neuropathological analyses. The general characteristics of Cohort 2 are listed in Table 2 and the Supporting Information Table S1. There were no differences in distributions of gender (*p* > 0.999), age (*p* = 0.701), tumour staging (*p* = 0.890) or postmortem interval (PMI, *p* = 0.738). CC patients were selected using clinical records data and following cachexia diagnostic criteria by Evans et al. 2008 [1] (see Section 2, Table 2 and the Supporting Information Table S1).

#### 6.2.1 Neuronal Count and Neuronal Density in the Cachectic Brain

**FIGURE 4:**
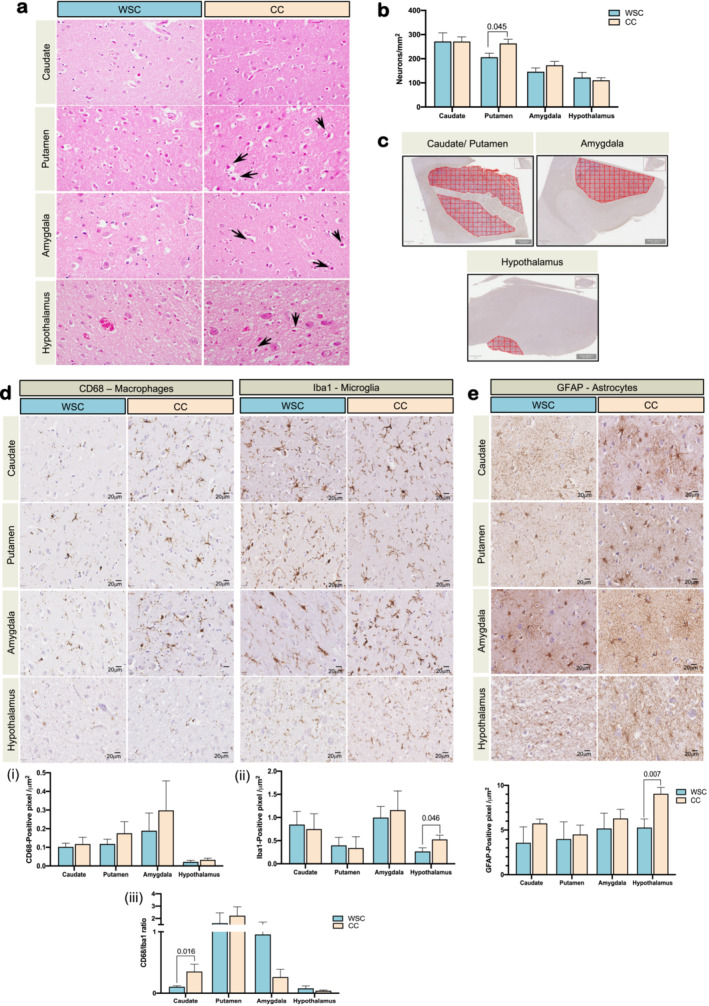
Neuropathological analyses in *postmortem* human brain reveals differences in cachexia. 400x magnification optic microscopy images. Significant differences between the groups were tested using unpaired *T*‐test. WSC, weight stable cancer, *n* = 6; CC: Cancer Cachexia, *n* = 10. (a) H&E staining in human brain, → showing altered neuronal structures; (b) Neuronal density (neurons/mm^2^) data in Cohort 2; (c) automated quantification in the regions of interest using Qupath v.0.1.2; (d) left panel: CD68 staining in ROIs; right panel: Iba1 staining in ROIs; lower panel: *T*‐test statistics of CD68 and Iba1 staining; (e) GFAP staining analyses in ROIs.

Cachectic cases H&E sections showed neurons with shrunken cytoplasm and dark, sometimes pyknotic nuclei (see Figure 4a, black arrows). Quantitatively, neuronal density (neuronal counts per area [mm^2^]) was significantly increased in the putamen of cachectic patients vs. weight stable controls (206 ± 16 neurons/mm^2^ WSC vs 263 ± 18 neurons/mm^2^ CC, *p* = 0.045) (Figure 4b and Extended Data S7). No significant difference in neuronal density was found in the caudate nucleus, amygdala or hypothalamus (*p* > 0.05) (Figure 4b and Extended Data S7).

#### 6.2.2 Hypothalamic Neuroglia Is Affected in Human Cancer Cachexia

Microglial phenotypes were assessed using Iba1 and CD68 markers. Iba1 labelling, considered a pan microglial marker, was higher in the hypothalamus of CC patients (0.53 ± 0.09 positivity), relative to WSC patients (0.27 ± 0.08 positivity) (*p* = 0.046, Figure 4d (ii) and Extended Data S8). Caudate nucleus, putamen and amygdala did not present differences in Iba1 staining between groups. Also, no differences were found for the CD68 marker, which is upregulated in actively phagocytic cells, in the brain ROI between WSC and CC (Figure 4d (i) and Extended Data S9). Nevertheless, CD68/Iba1 ratio, combining the quantitative measurements of CD68 and Iba1, was increased in the caudate nucleus of CC (0.35 ± 0.12) as compared with WSC (0.10 ± 0.02), demonstrating an increase in lysosomal and phagocytic activity relative to overall microglial/macrophage density (*p* = 0.016, Figure 4d (iii) and Extended Data S10). Glial fibrillary acidic protein (GFAP) is a specific marker for astrocytes that presented different patterns between groups. Increased perivascular GFAP was found in CC vs. WSC (Extended Data S11). Quantitatively, hypothalamic GFAP staining was increased in CC (9.06 ± 0.72 GFAP+) relative to WSC (5.27 ± 0.97 GFAP+) (*p* = 0.007, Figure 4e and Extended Data S12). No differences were found in the caudate nucleus, putamen and amygdala (Figure 4e and Extended Data S12).

#### 6.2.3 mTOR Pathway Is Involved in Cachexia‐Related Neuroinflammation

**FIGURE 5:**
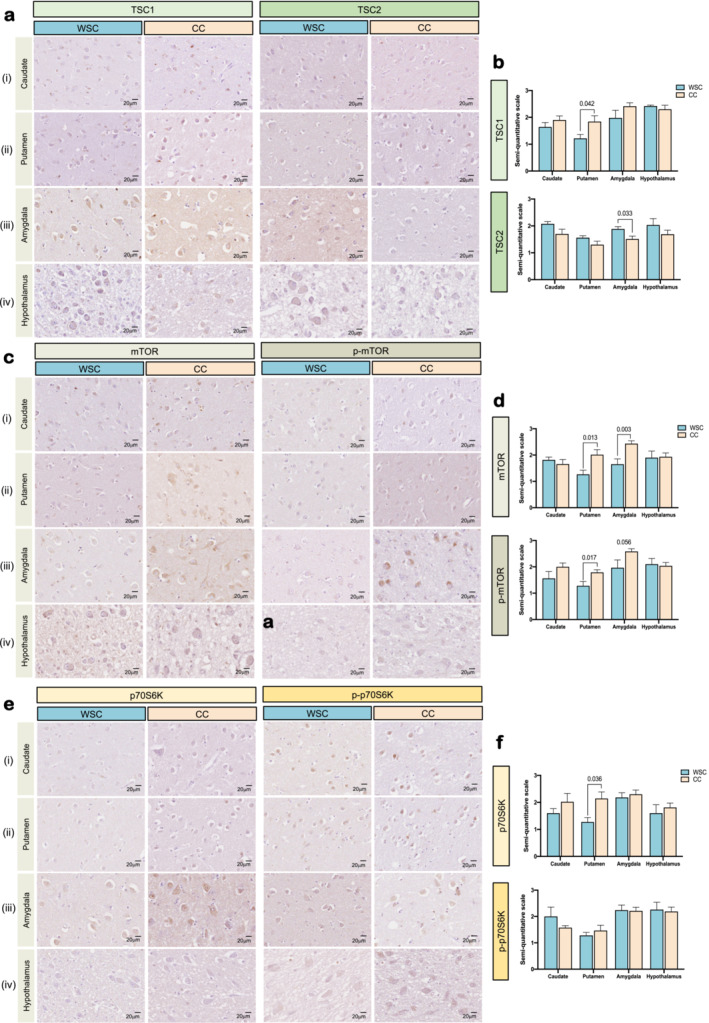
Semiquantitative mTOR pathway analyses in human brain tissue. 400x magnification optic microscopy images. Data expressed as mean ± SEM. Significant differences between the groups were tested using unpaired *T‐*test. WSC, weight stable cancer, *n* = 4–6; CC, cancer cachexia, *n* = 6–10. (a, b) Upstream mTOR pathway‐TSC1 and TSC2 staining and statistics in ROIs; (c, d) mTOR pathway core‐ mTOR and p‐mTOR staining and statistics in ROIs; (e, f) downstream mTOR pathway‐p70S6K and p‐p70S6K staining and statistics in ROIs; mTOR, mechanistic targeting pathway of rapamycin; p70S6K, ribosomal protein S6 kinase; p‐p70S6K, phosphorylated p70S6K; p‐mTOR, phosphorylated mTOR; TSC1, hamartin; TSC2, tuberin.

Given the importance of mTOR for immune cell responses in the CNS and to provide a global measure for mTOR activity, the distribution and extent of a subset of core upstream (TSC1 and TSC2) and downstream (mTOR, phospho‐mTOR, p70S6K and phospho‐p70S6K) mTOR‐related pathway proteins were assessed (Figure 5a–f). Analyses were performed on the caudate nucleus, putamen, amygdala and hypothalamus, to assess a possible contribution of the pathway to the changes observed in the CNS due to the presence of cachexia.

Semiquantitative analyses of upstream mTOR pathway markers showed that TSC1 expression is higher in the putamen of CC compared to WSC (*p* = 0.042, Figure 5a **(ii)** and Figure 5b). For TSC2, a lower content was observed in the amygdala of CC vs. WSC patients (*p* = 0.033, Figure 5a **(iii)** and Figure 5b). No other differences were found in TSC1 or TSC content in the other ROIs (*p* > 0.05, Figure 5a,b). Total mTOR content was higher in the putamen (*p* = 0.013, Figure 5c (ii) and Figure 5d) and in the amygdala (*p* = 0.003, Figure 5c (iii) and Figure 5d) of CC, compared with WSC. In addition, phospho‐mTOR staining was significantly increased in the putamen of cachectic patients (*p* = 0.017, Figure 5c (ii) and Figure 5d), and statistical tendence implying higher content was detected in the amygdala of CC relative to WSC (*p* = 0.056, Figure 5c (iii) and Figure 5d). Considering downstream mTOR pathway markers, p70S6K was found to be increased in the putamen of CC relative to WSC (*p* = 0.036, Figure 5e (ii) and Figure 5f). No other significant differences were found for p70S6K or phosphor‐p70S6K between groups or ROI (*p* > 0.05) (Figure 5e–f).

## 7 Discussion

This original in vivo neuroimaging study reveals altered morphology, connectivity, and function in the brain of CC patients in comparison with weight‐stable cancer patients. The whole‐brain exploratory VBM data and SVC‐based analyses in a priori selection of ROIs demonstrated specific grey matter volume (GMV) alterations in several regions, such as the orbitofrontal cortices, caudate, putamen, superior temporal gyrus and operculum (Figure 2, Extended Data S2). These structures are classically involved in the processing of visceral afferent information (interoception), reward‐motivated behaviour, stress‐related behaviour, emotional state, and food intake, as well as in the interpretation of gustatory, olfactory and visual stimuli or food cue‐reactivity [23, 24], ^S27–28^ which are directly associated with weight loss, protein intake or body composition ^S29^. In fact, many of these structures showing altered volume in CC are involved in the reward circuit and in charge of responding to food stimuli, by receiving the metabolic signals of hunger or satiety [18], ^S30^. On the other hand, increased GMV in CC could indicate acute inflammation of brain structures, concomitant with or resulting from systemic inflammation associated with cachexia. In support of this idea, several mice models of cachexia demonstrate an increased content of hypothalamic TNFα and IL1 [4], ^S31^. Moreover, reduced operculum and temporal gyrus GMV could be a sign of atrophy resulting from the syndrome. The observed alterations in the GM of cachectic patients are compatible with the effect described for different acute and short‐term diseases, such as those of patients suffering from traumatic brain injury (TBI) [25]. The literature provides evidence that neuronal loss does not occur in the early stages of chronic disease and shows that TBI patients do not present atrophy in the semi‐acute phase [25]. However, structures with increased volumes (orbitofrontal cortex, caudate nucleus and putamen) may suffer atrophy induced by uncontrolled inflammation, as described for patients with obstructive sleep apnea (OSA) [26]. Hypoxic events cause an increase in the permeability of the blood–brain barrier [27], which leads to enlarged GM in OSA patients caused by reversible or short‐term vasogenic cerebral edema [26]. In those patients, surgical treatment was able to counteract the severity of the disease and concomitant systemic inflammation, with subsequent recovery of the initially altered brain structures [26], ^S32^. Moreover, in accordance with our results, several studies in Type 1 and Type 2 diabetes have reported increases or decreases in different frontal regions associated with emotional and cognitive processing, postulating that insulin resistance (which can induce neuroinflammation through the oxidative stress pathway) may be one of the causes inducing such alterations in the metabolic syndrome [28, 29], ^S33^.

Altogether, our VBM data demonstrate that cachexia affects GM morphology within regions that may play an important role in the patient's food intake and quality of life. Given the composition of GM and WM, we believe that the acute inflammation and neuroinflammatory response resulting from cachexia rapidly affect the neuronal cell bodies and glial cells constituting the GM ^S34^ but show little impact on the components of WM. In this regard, we were unable to find any evidence that cachexia alters any aspect of the WM. We report no differences in GM volumes or/and GM SVC‐based analyses (VBM, Extended Data S3), as well as no alterations in fractional anisotropy (DTI, Extended Data S4) similar to those described in long‐term and neurodegenerative diseases such as multiple sclerosis [30].

To examine whether the brain alterations observed are concomitant with and/or result from systemic inflammation, a hallmark of the cachexia syndrome, we carried out correlation analyses between anthropometric data, circulating inflammatory markers and the GMV of the ROIs (Figure 2c). Our data revealed that increased and decreased GMV were negatively correlated with current BM in CC patients, indicating that brain morphological changes may be driving the emaciation resulting from cachexia [31], ^S35^. In the cachectic group, a negative correlation between IL8 content and caudate nucleus volume was found. IL8 levels have been associated with major depressive disorders and sickness behaviour [32], ^S36^. Moreover, several studies in animal models with Alzheimer's disease have shown that higher IL8 content promotes smaller volumes of GM in the hippocampus of monkeys, as well as brain tissue damage in rodents, through neurotoxic action by augmenting the transendothelial migration of neutrophils, increasing the release of reactive oxygen species (ROS) and the production of neutrophil‐mediated proteases [33], ^S37^.

To conclude our in vivo neuroimaging study, we evaluated the effect of cachexia on FC using rsfMRI. CC patients had 20 negative and 5 positive significant different connections compared to the WSC group (Figure 3). Those changes were observed in fundamental regions related to body weight regulation and emotional state (insula, caudate nucleus, putamen, nucleus accumbens, temporal gyrus and temporo‐occipital gyrus, among others; Extended Data S5). In overnight fasted healthy individuals, Cornier et al. showed that there is a positive activation of different regions related to the perception of food signals and greater motivation to eat (such as the insula, temporal visual cortex, parietal cortex, ventral striatum, posterior cingulate, hippocampus, sensory cortex and lateral prefrontal cortex) [34]. Nevertheless, our fasted CC group showed reduced connectivity in these same regions, suggesting that cachectic patients have a reduced impulse to eat even in the postnight fasting state, which may be associated with the anorexia characteristic of the syndrome. In line with our data, a reduction in the efficiency of the insula, the temporal region and the cingulate cortex has been reported in patients with early‐stage anorexia nervosa [35], ^S38^. Furthermore, in the reward circuits (corpus striatum – caudate nucleus and putamen), the response to hunger or satiety does not differ in anorexia nervosa patients ^S22^. In contrast, obese individuals showed hyperactivity of the insula in response to food stimuli [36]. The insula plays an important role in somatosensory, visceral sensory and visceral motor functions and is highly sensitive to peripheral signals [37]. In an fMRI study carried out on with adults with leptin deficiency, it was shown that the deficiency of this hormone is associated with greater activation of the insula in response to visual food stimuli [38]. The mechanism involved is unknown, but we propose that the low leptin content present in cachectic patients could indicate an inability to stimulate the insula (note that this is a region that has shown morphological and functional alterations associated with the syndrome). In the present in vivo study, we also analysed several brain metabolites on the hypothalamus assessed by MRS. However, we were not able to identify any differences in hypothalamic levels of metabolites (Extended Data S6). Considering the difficulty to obtain quality images of a small region (hypothalamus), we suggest that future studies should increase the number of samples. In 2020, a 1H‐MRS study in rodents showed a significant depletion of choline, as well as an increase in glutamine in the cachectic brains [39]. Given that choline is essential for the synthesis of the neurotransmitter acetylcholine, a decrease in choline in the context of cachexia could lead to changes in cholinergic activity, which plays a key role in modulating inflammation and appetite [40].

To confirm our in vivo neuroimaging results, we proceeded to carry out neuropathological analyses on ROIs in postmortem tissue provided by the Oxford Brain Bank (UK). The postmortem data revealed that cachectic patients presented neurons with retracted cytoplasm and dark nuclei, sometimes appearing pyknotic, as assessed by H&E staining (Figure 4a). These changes have been also observed in neurodegenerative diseases, where altered processes (oxidative stress, increased neurotoxic reagents or autophagy) trigger disturbances in neuronal function and structure, leading to neuronal death ^S39^. During systemic inflammation, peripheral signals integrated into the CNS such as CRP or inflammatory cytokines can induce the observed neural alterations ^S40^. In addition, we observed changes in neuroglia profile in cachectic patients. CC presented a higher CD68/Iba1 ratio in the caudate nucleus, what may indicate that cachexia leads towards a microglia profile with increased phagocytic activity (CD68) in relation to the total microglia density (Iba1) in that region (Figure 4d). Microglia can orchestrate a powerful CNS inflammatory response, and alterations suffered by this population are directly associated with the progression of some pathologies [41], ^S41^. Particularly in cachexia, it has been postulated that systemic inflammation causes hyperactivation of the microglia which perpetuates a neuroinflammatory scenario in which microglia shifts towards neurotoxic profile with phagocytic potential and enhanced ability to produce more pro‐inflammatory mediators other than those released systemically [10], ^S42^. Recent studies in rodents with pancreatic adenocarcinoma have shown an increase in active microglia (characterised by retracted processes and increase in soma size) in the median eminence and in the arcuate nucleus of the hypothalamus of cachectic animals [9]. Moreover, Burfeind et al. (2020) demonstrated that balanced microglia may offer a protective effect in cachexia, since complete depletion of this population aggravates muscle catabolism [9]. Our data also show that the astrocyte GFAP marker label is qualitatively higher in the perivascular regions (Extended Data S11) and quantitatively increased in the hypothalamus of CC (Figure 4e and Extended Data S12), in relation to WSC. GFAP positivity increase has been well described in neurodegenerative diseases, such as Alzheimer's, in which astrocytes have been postulated as possible therapeutic targets [42]. Pancreatic cancer models show that cachexia is associated with neural invasion, and a direct relationship has been observed between neural invasion and astrocyte activation in the spinal cord [43]. An anticachexic effect was also reported when astrocyte activation was suppressed [43]. The involvement of astrocytes in the pathology of cachexia may be associated with the loss of normal homeostatic functions and the gain of toxic functions [44], ^S43^.

To shed light on possible mechanisms associated with neuroinflammation and disrupted neuroglia profile that we observed in our postmortem study, we evaluated the mTOR pathway (Figure 5), given its crucial role in brain physiology and pathophysiology [15]. Cachectic patients showed altered upstream marker content of the mTOR pathway proteins (TSC1 and TSC2) in the putamen (increased TSC1) and the amygdala (decreased TSC2) (Figure 5a (ii‐iii) and Figure 5b). TSC1 and TSC2 form a complex, and both proteins are necessary for inhibitory function upon mTOR [45]. In Alzheimer's disease, TSC1 aggregates form in the absence of TSC2 in the CA1 region of the hippocampus, leading to increased inflammation, altered autophagy, β‐amyloid accumulation and cell death via mTOR [46]. In CC, we observed that TSC1/TSC2 alterations lead to greater inflammation with increased mTOR and p‐mTOR content in the putamen and the amygdala (Figure 5c (ii‐iii) and Figure 5d). Also, increased p70S6K was observed in the putamen of CC (Figure 5e (ii) and Figure 5f). Our data suggest that CC shows higher activation of the mTOR pathway in the putamen and in the amygdala, which possibly, a severe negative impact leading to synaptic dysfunction, inhibition of neurogenesis, priming of microglia or neural death [15].

To the best of our knowledge, the results obtained in the present study (in vivo neuroimaging and postmortem neuropathology) are the first to describe changes in brain morphology and function as induced by cachexia in cancer patients. We demonstrated that human cancer cachexia alters GM morphology and brain FC and affects neuronal density, microglial and astrocyte profile. These changes were concomitant with mTOR pathway‐protein expression and phosphorylation alterations (see Graphical Abstract).

Limitations of our study should be acknowledged. It remains to be assessed whether the changes we report are directly involved in the cause of cachexia and its systemic effects or are a consequence of the syndrome. In the present study, we evaluated CNS alterations associated with cachexia solely in colorectal cancer patients (Cohort 1, in vivo neuroimaging) or gastrointestinal carcinoma cadaver samples (Cohort 2, postmortem neuropathological analyses). Future longitudinal studies are required to evaluate whether these CNS alterations could lead to a worse prognosis and quality of life.

## Conflicts of Interest

The authors declare no conflicts of interest.

## Supporting information


**Data S1.** Extended Data 1 Total brain volume.
Extended Data 2 Grey matter analysis in WSC and CC patients.
Extended Data 3 White matter analysis in WSC and CC patients.
Extended Data 4 Diffusion Tensor Imaging (DTI) ‐ Differences in fractional anisotropy values.
Extended Data 5 Resting state functional MRI (rsfMRI) analysis in WSC and CC patients.
Extended Data 6 Data obtained using magnetic resonance spectroscopy (MRS) focused on the hypothalamus.
Extended Data 7 Neuronal density (neurons/mm2) in human brain tissue.
Extended Data 8 Quantitative data of Iba1 staining.
Extended Data 9 Quantitative data of CD68 staining.
Extended Data 10 Quantitative data of CD68/Iba1 ratio.
Extended Data 11 Qualitative analysis of perivascular GFAP staining.
Extended Data 12 Quantitative data of GFAP staining.

**Data S2.** Supplementary methods 1. MRI acquisition.
Supplementary Methods S2. DTI data processing.
Supplementary Methods S3. DTI data processing.
Supplementary Methods S4. rsfMRI data processing.
Supplementary Methods S5. MRS quantification data processing.
Supplementary Methods S6. Immunohistochemistry (IHC) protocol.
Supplementary Methods S7. Regional analysis.
Supplementary Methods S8. Neuroimaging statistical analysis.
Supplementary Table S1 Case demographics of postmortem human tissue selected.
Supplementary Table S2 Optimised antibodies.
Supplementary Table S3 Pixel counting qupath algorithms.
Supplementary Figure S1 TSC1 semiquantitative scale per region of interest.
Supplementary Figure S2 TSC2 semiquantitative scale per region of interest.
Supplementary Figure S3 mTOR semiquantitative scale per region of interest.
Supplementary Figure S4 phospho‐mTOR semiquantitative scale per region of interest.
Supplementary Figure S5 p70S6K semiquantitative scale per region of interest.
Supplementary Figure S6 phospho‐p70S6K semiquantitative scale per region of interest.

**Data S3.** Supporting Information.
