# Supplementary material for: Cachexia Alters Central Nervous System Morphology and Functionality in Cancer Patients
Source: J Cachexia Sarcopenia Muscle. 2025 Feb 17;16(1):e13742. doi: 10.1002/jcsm.13742 (PMC11832348; doi:10.1002/jcsm.13742)
Supplement: Supplementary file 1 — Data S1. Extended Data 1 Total brain volume. Extended Data 2 Grey matter analysis in WSC and CC patients. Extended Data 3 White matter analysis in WSC and CC patients. Extended Data 4 Diffusion Tensor Imaging (DTI) ‐ Differences in fractional anisotropy values. Extended Data 5 Resting state functional MRI (rsfMRI) analysis in WSC and CC patients. Extended Data 6 Data obtained using magnetic resonance spectroscopy (MRS) focused on the hypothalamus. Extended Data 7 Neuronal density (neurons/mm2) in human brain tissue. Extended Data 8 Quantitative data of Iba1 staining. Extended Data 9 Quantitative data of CD68 staining. Extended Data 10 Quantitative data of CD68/Iba1 ratio. Extended Data 11 Qualitative analysis of perivascular GFAP staining. Extended Data 12 Quantitative data of GFAP staining. [file JCSM-16-e13742-s002.docx]

**Extended data**

**Extended Data 1** Total brain volume

| **Total Volume** | **WSC** | **CC** | ***p-value*** |
| --- | --- | --- | --- |
| GM (mL) | 612.0 ± 14.3 | 605.6 ± 10.9 | 0.735 |
| WM (mL) | 382.3 ± 11.0 | 389.2 ± 13.2 | 0.692 |
| CSF (mL) | 265.4 ± 24.9 | 270.0 ± 31.8 | 0.910 |
| TBV (mL) | 994.3 ± 21.7 | 994.7 ± 22.7 | 0.989 |
| TIV (mL) | 1260 ± 28.6 | 1265 ± 43.0 | 0.922 |

Data expressed as mean ± SEM. WSC: Weight stable cancer; CC: Cancer Cachexia; mL: milliliters; GM: gray matter; WM: White matter; CSF: Cerebrospinal fluid; TBV: Total brain volume; TIV: Total intracranial volume. Significant differences were tested using unpaired T-test.

**Extended Data 2** Gray matter analysis in WSC and CC patients


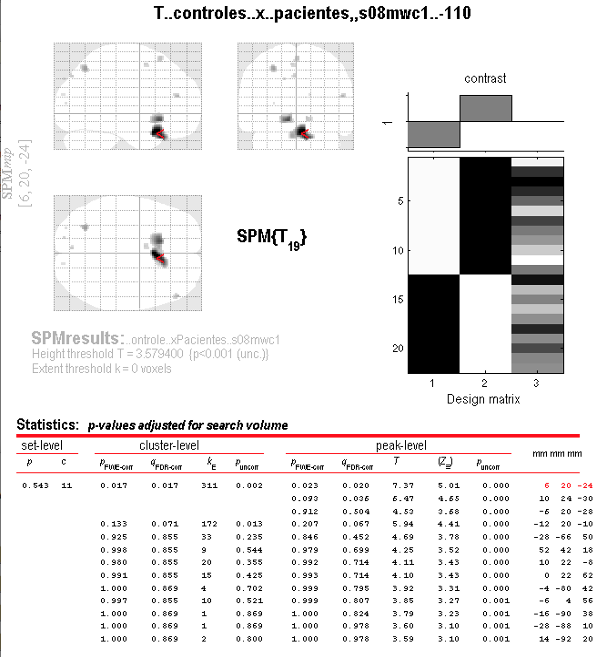

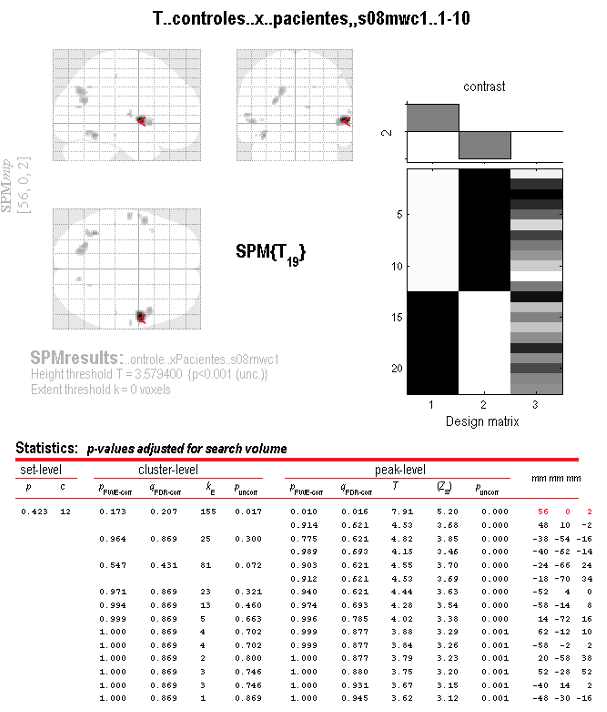


Left panel represents increased regions in CC compared to WSC patients. Right panel represents the decreased regions in the CC group.

Significant p-value at pFWE<0.05.

**Extended Data 3** White matter analysis in WSC and CC patients


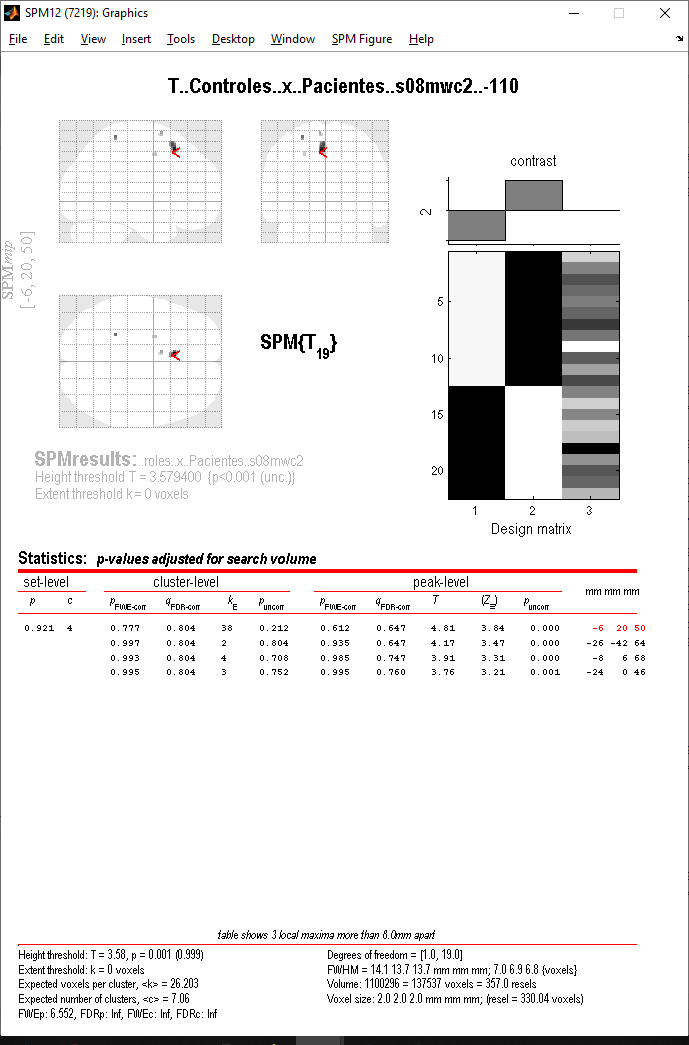

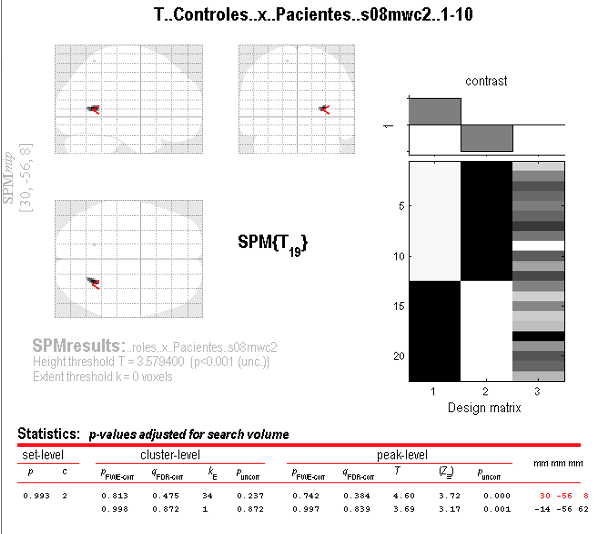


Left panel represents increased regions in CC compared to WSC patients. Right panel represents the decreased regions in the CC group.

Significant p-value at pFWE<0.05.

**Extended Data 4** Diffusion Tensor Imaging (DTI) - Differences in fractional anisotropy values


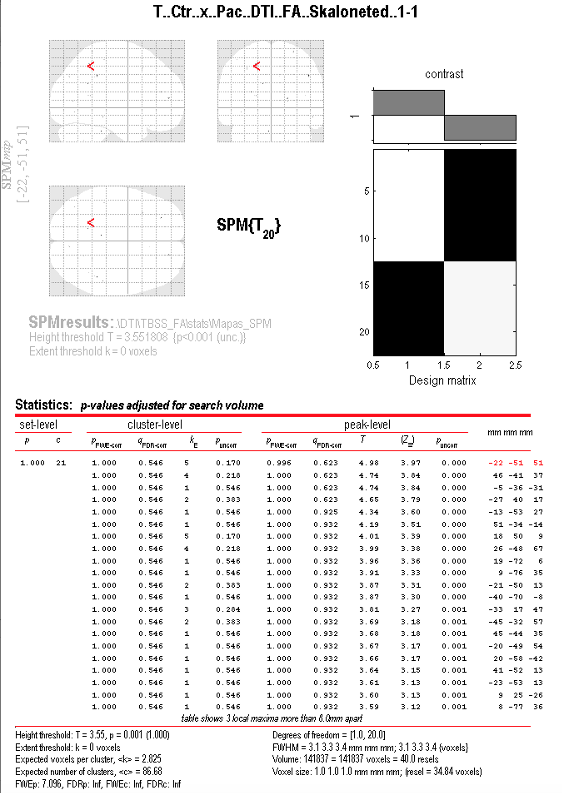

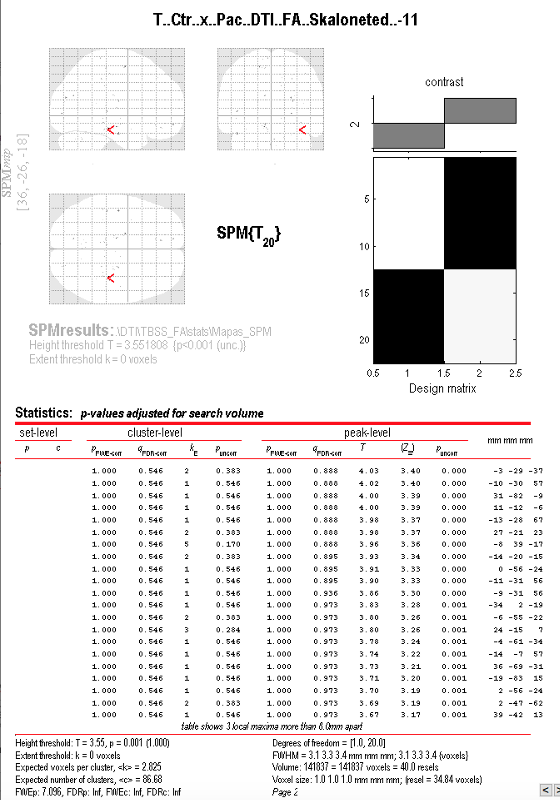


Left panel represents increased regions in CC compared to WSC patients. Right panel represents the decreased regions in the CC group.

Significant p-value at pFWE<0.05.

**Extended Data 5** Resting state functional MRI **(**rsfMRI) analysis in WSC and CC patients

| **ROIs** | **Negative FC**  **[Nb voxels (x,y,z)]** | **pFDR** | **Positive FC**  **[Nb voxels (x,y,z)]** | **pFDR** |
| --- | --- | --- | --- | --- |
| Salience network | | | | |
| Insula L | [217 (-26, -42, +36)]: Cingulate Gyrus Post. | 0.0047 |  |  |
|  | [183 (-08, +34, +20)]: Paracingulate Gyrus R/L | 0.0060 |  |  |
|  | [110 (+12, +08, +30)]: Cingulate Gyrus Ant.; Paracingulate Gyrus R/L | 0.0392 |  |  |
| Insula R | [114(-14, -42, +50)]: Postcentral Gyrus L; Precuneous Cortex | 0.0381 |  |  |
|  | [111 (-02, +14, +52)]: Paracingulate Gyrus L; Frontal Gyrus Sup. L | 0.0381 |  |  |
| Network Salience-Insula R | [150 (-36, +24, -06)]: Insular cortex L; Orbitofrontal Cortex L; Frontal Operculum Cortex L | 0.0311 |  |  |
| Network Salience-RPFC R | [158 (+22, -82, -28)]: Cerebellum R | 0.0105 |  |  |
| Subcortical Areas | | | | |
| Nucleus Accumbens R | [189 (+32, -70, +24)]: Lateral Occipital Cortex Sup. R | 0.0017 |  |  |
|  | [185 (+26, -68, +44)]: Lateral Occipital Cortex Sup. R | 0.0017 |  |  |
|  | [101 (-20, -80, +20)]: Lateral Occipital Cortex Sup. and Inf. L | 0.0260 |  |  |
|  | [84 (-36, -46, +50)]: Parietal Lobule Sup. L; Supramarginal Gyrus Post. L. | 0.0385 |  |  |
|  | [101 (-20, -80, +20)]: Lateral Occipital Cortex Sup. L; Parietal Lobule Sup. L | 0.0260 |  |  |
| Putamen L | [238 (-06, -46, +54)]: Precuneous Cortex; Postcentral Gyrus R/ L | 0.0011 | [144 (-16, -84, -06)]: Occipital Fusiform Gyrus L; Lingual gyrus L; Intracalcarine Cortex L | 0.0107 |
|  | [104 (+60, -34, +52)]: Supramarginal Gyrus Ant. e Post. R; Postcentral Gyrus R | 0.0304 | [91 (-08, -56, -14): Cerebelum, Vernis | 0.0379 |
|  |  |  | [83 (+34, -82, -14)]: Lateral Occipital Cortex Inf. R; Occipital Fusiform Gyrus R | 0.0420 |
| Posterior Parahippocampal Gyrus R |  |  | [191 (+42, -76, +18)]: Lateral Occipital Cortex, Sup. and Inf. R | 0.0019 |
| Temporal Cortical areas | | | | |
| Superior Temporal Gyrus, Ant. R | [170 (-52, -04, +54): Precentral Gyrus L; Frontal Gyrus Mid. L | 0.0099 |  |  |
| Superior Temporal Gyrus, Post. L | [130 (-58, -20, -18): Temporal Gyrus Mid. Post. L; Temporal Gyrus Inf. Post. L | 0.0103 |  |  |
| Middle Temporal Gyrus, Ant. R | [113 (+24, -56, +04)]: Lingual Gyrus R; Precuneous Cortex | 0.0480 |  |  |
| Middle Temporal Gyrus, temporooccipital L | [133 (+40, +20, +30)]: Frontal Gyrus Mid. R; Frontal Gyrus Inf. pars opercularis and triangularis R | 0.0228 |  |  |
| Inferior Temporal Gyrus, temporooccipital R | [202 (+14, +48, +40)]: Frontal Pole R/L; Frontal Gyrus Sup. L/R | 0.0058 |  |  |
|  | [155 (-10, +12, +64)]: Frontal Gyrus Sup. L | 0.0100 |  |  |
| Inferior Temporal Gyrus, temporooccipital L |  |  | [131 (+28, -40, +18)] White matter Callosal body R | 0.0356 |

R: Right; L: Left; Sup.: Superior; Inf.: Inferior; Post.: Posterior.; Ant.: Anterior; RPFC: Right Prefrontal Cortex. Coordinates (x,y,y): voxel coordinates of maximum statistical significance within each cluster; pFDR: corrected for multiple comparison using false discovery rate (FDR). Significant differences between groups were tested using the unpaired t-test.

**Extended Data 6** Data obtained using magnetic resonance spectroscopy (MRS) focused on the hypothalamus

| **Ratios** | **WSC** | **CC** | ***p-value*** |
| --- | --- | --- | --- |
| **Glu/Cr** | 0.939 ± 0.08 | 1.053 ± 0.15 | 0.553 |
| **Glx/Cr** | 1.567± 0.12 | 1.833 ± 0.17 | 0.294 |
| **NAA/Cr** | 0.788 ± 0.09 | 0.977 ± 0.10 | 0.214 |
| **ml/Cr** | 1.022 ± 0.11 | 1.039 ± 0.12 | 0.921 |
| **GPC+PCh/Cr** | 0.352 ± 0.03 | 0.355 ± 0.01 | 0.931 |

Data expressed as mean ± SEM. WSC: Weight stable cancer; CC: Cancer Cachexia; Cr: creatine; Glu: glutamate; Glx: sum of glutamate and glutamine; NAA: N-acetylaspartate; mI: myoinositol. GPC + PCh: sum of glycerophosphocholine (GPC) and phosphocholine (PCh). Significant differences were tested using unpaired T-test.

**Extended Data 7** Neuronal density (neurons/mm2) in human brain tissue.

| **Neurons/mm^2^** | **WSC** | **CC** | ***p-value*** |
| --- | --- | --- | --- |
| **Caudate** | 271 ± 35 | 274 ± 19 | 0.994 |
| **Putamen** | 206 ± 16 | 263 ± 18 | **0.045** |
| **Amygdala** | 146 ± 15 | 173 ± 16 | 0.286 |
| **Hypothalamus** | 122 ± 21 | 110 ± 10 | 0.599 |

Data expressed as mean ± SEM. WSC: Weight stable cancer; CC: Cancer Cachexia. Significant differences were tested using unpaired T-test.

**Extended Data 8** Quantitative data of Iba1 staining

| **Iba1** | **WSC** | **CC** | ***p-value*** |
| --- | --- | --- | --- |
| **Caudate** | 0.85 ± 0.28 | 0.75 ± 0.33 | 0.829 |
| **Putamen** | 0.39 ± 0.17 | 0.34 ± 0.24 | 0.859 |
| **Amygdala** | 0.99 ± 0.24 | 1.16 ± 0.41 | 0.757 |
| **Hypothalamus** | 0.27 ± 0.08 | 0.53 ± 0.09 | **0.046** |

Data expressed as mean ± SEM. WSC: Weight stable cancer; CC: Cancer Cachexia. Significant differences were tested using unpaired T-test.

**Extended Data 9** Quantitative data of CD68 staining

| **CD68** | **WSC** | **CC** | ***p-value*** |
| --- | --- | --- | --- |
| **Caudate** | 0.10 ± 0.02 | 0.12 ± 0.04 | 0.746 |
| **Putamen** | 0.12 ± 0.03 | 0.18± 0.06 | 0.483 |
| **Amygdala** | 0.19 ± 0.09 | 0.29 ± 0.16 | 0.653 |
| **Hypothalamus** | 0.02 ± 0.008 | 0.03 ± 0.009 | 0.446 |

Data expressed as mean ± SEM. WSC: Weight stable cancer; CC: Cancer Cachexia. Significant differences were tested using unpaired T-test.

**Extended Data 10** Quantitative data of CD68/Iba1 ratio

| **CD68/Iba1 Ratio** | **WSC** | **CC** | ***p-value*** |
| --- | --- | --- | --- |
| **Caudate** | 0.10 ± 0.02 | 0.35 ± 0.12 | **0.016** |
| **Putamen** | 1.61 ± 0.84 | 2.22 ± 0.72 | 0.445 |
| **Amygdala** | 0.96 ± 0.74 | 0.26 ± 0.13 | 0.247 |
| **Hypothalamus** | 0.08 ± 0.04 | 0.04 ± 0.01 | 0.254 |

Data expressed as mean ± SEM. WSC: Weight stable cancer; CC: Cancer Cachexia. Significant differences were tested using unpaired T-test.

**Extended Data 11** Qualitative analysis of perivascular GFAP staining

Optical microscopy images with 100x magnification. WSC: Weight stable cancer; CC: Cancer Cachexia

**Extended Data 12** Quantitative data of GFAP staining

| **GFAP** | **WSC** | **CC** | ***p-value*** |
| --- | --- | --- | --- |
| **Caudate** | 3.57± 1.78 | 5.74 ± 0.48 | 0.136 |
| **Putamen** | 3.99 ± 1.92 | 4.50 ± 1.05 | 0.801 |
| **Amygdala** | 5.17± 1.72 | 6.29 ± 1.03 | 0.606 |
| **Hypothalamus** | 5.27± 0.97 | 9.06± 0.72 | **0.007** |

Data expressed as mean ± SEM. WSC: Weight stable cancer; CC: Cancer Cachexia. Significant differences were tested using unpaired T-test.
