# Supplementary material for: Cachexia Alters Central Nervous System Morphology and Functionality in Cancer Patients
Source: J Cachexia Sarcopenia Muscle. 2025 Feb 17;16(1):e13742. doi: 10.1002/jcsm.13742 (PMC11832348; doi:10.1002/jcsm.13742)
Supplement: Supplementary file 2 — Data S2. Supplementary methods 1. MRI acquisition. Supplementary Methods S2. DTI data processing. Supplementary Methods S3. DTI data processing. Supplementary Methods S4. rsfMRI data processing. Supplementary Methods S5. MRS quantification data processing. Supplementary Methods S6. Immunohistochemistry (IHC) protocol. Supplementary Methods S7. Regional analysis. Supplementary Methods S8. Neuroimaging statistical analysis. Supplementary Table S1 Case demographics of postmortem human tissue selected. Supplementary Table S2 Optimised antibodies. Supplementary Table S3 Pixel counting qupath algorithms. Supplementary Figure S1 TSC1 semiquantitative scale per region of interest. Supplementary Figure S2 TSC2 semiquantitative scale per region of interest. Supplementary Figure S3 mTOR semiquantitative scale per region of interest. Supplementary Figure S4 phospho‐mTOR semiquantitative scale per region of interest. Supplementary Figure S5 p70S6K semiquantitative scale per region of interest. Supplementary Figure S6 phospho‐p70S6K semiquantitative scale per region of interest. [file JCSM-16-e13742-s001.docx]

**Supplementary information**

**Supplementary Methods 1. MRI Acquisition**

VBM-Morphological brain data were acquired using a T1-weighted images with the following parameters: repetition time (TR)= 7ms; echo time (TE) = 3.2ms; number of excitations (NEX) = 1; field of view (FOV) = 240x240x180 mm; slice thickness = 1mm, no gaps, matrix = 240x240 pixels; and voxel size = 1x1x1 mm, resulting in 180 slices covering the whole brain. The estimated time for the acquisition of the sequences was approximately 30 minutes.

We acquired DTI data using a sequence of T2-weighted images without diffusion gradient (b=0 s/mm2), gradient diffusion images (b= 1000S/mm²) obtained in 33 non-collinear directions. Data acquisition parameters were as follows: TR=8.7ms, TE= 65ms, flip angle=90, FOV = 256x256x256mm, slice thickness = 2mm, no gaps, number of averages: 3, acquisition matrix = 128 × 128 pixels, resulting in voxels with an isotropic size of 2x2x2mm, with 50 sections covering the whole brain. The estimated time for the acquisition of the sequences was approximately 20 minutes.

Resting state fMRI data acquisition employed a T2-weighted echoplanar image sequence and the following parameters: TR= 2000ms, TE=30ms, inclination angle= 80, FOV = 240x240mm, with an interval between slices of 0.5mm, acquisition matrix = 80x80 pixels. Resulting in 32 slices, with 4mm thick sections. To perform the fMRI, the patients were informed to keep their eyes open and look at a fixed point, without any external stimuli. The estimated sequence acquisition time was approximately 10 minutes.

MRS focused on the hypothalamic region was performed using the PRESS technique (Point Resolved single voxel) with TE = 35ms, TR = 4000ms and 160 repetitions. 16 spectra without water suppression were also acquired for reference purposes and error correction of spurious currents. The nominal voxel size of the spectrum was 8cm^3^ (2x2x2cm^3^), located at the height of the hypothalamus. To reduce the chemical shift effect, four saturation bars were placed within the limits of the water-voxel overlap and N-acetylaspartate (NAA). The estimated time for acquisition of the sequences was approximately 8 minutes.

**Supplementary Methods 2. DTI data processing**

Initially, all anatomical images were reoriented to match the anterior commissure's coordinates with the origin (0, 0, 0), and their orientation was aligned with the Montreal Neurological Institute (MNI) space. Subsequently, the images were segmented into gray matter (GM), white matter (WM) and cerebrospinal fluid (CSF) partitions using the unified segmentation procedure described by Ashburner and Friston ^S44^. The Diffeomorphic Anatomical Registration Through Exponentiated Lie Algebra (DARTEL) algorithm was applied for spatial normalization, maximizing sensitivity and accuracy by using an asymmetric T1-weighted template derived from participants' structural images instead of a standard template ^S45^. The fully normalized images were resliced to a final voxel size of 2x2×2 mm³ using trilinear interpolation. An additional "modulation" step involved multiplying each spatially normalized GM and WM image by its relative volume before and after normalization, preserving the total amount of GM and WM in each voxel. Finally, the resulting GM and WM images were smoothed with an 8-mm isotropic kernel at full width at half maximum (FWHM) to ensure data followed a normal distribution for subsequent statistical parametric tests.

**Supplementary Methods 3. DTI data processing**

The distortions caused by the large diffusion-sensitizing gradients and motion were corrected, using as a guide the b0 volume. Afterward, an automated procedure for segmenting and making brain and nonbrain tissues was applied; then, eigenvalues and eigenvectors were estimated in order to compute FA values, which were used in the present study. Then, we used Tract-Based Spatial Statistics (TBSS) on the FSL to process fiber tracts. TBSS involves several preprocessing steps before final analyses. First, FA maps of all subjects were aligned to each other based on free-form deformations and B-spline nonlinear registration approach ^S46^. The target image was affine-aligned into MNI standard space, and every image was transformed into 1 × 1 × 1 mm^3^ MNI standard space by combining the obtained linear and nonlinear transformation parameters. Next, the mean FA image was computed and thinned to create a mean FA (skeletonization). Then, each subject's aligned FA data were then projected onto this skeleton. Such procedure is taken by searching the highest FA values of the subject's perpendicular tract direction within mean FA skeleton. The data were then exported to the SPM12, to conduct the between-groups comparative statistical analysis.

**Supplementary Methods 4.** **rsfMRI data processing**

The anatomical component-based noise correction algorithm (aCompCor) method ^S47^ was used to denoise the potential confounding effect of the functional data from white matter and cerebrospinal areas (5 components each), estimated subject-motion parameters (12 components), scrubbing (100 components), and session effect (2 components), and simultaneously, the temporal band-pass filter was applied to remove frequencies below 0.008Hz from the BOLD signal as a single regression step ^S48^.

**Supplementary Methods 5.** **MRS quantification data processing**

An unsuppressed water signal was used as an internal reference. To ensure the quality and accuracy of the measurements, all spectra with a Cramer-Rao lower limit values (CRLB) of less than 20% ^S49^, low spectral resolution [width at half height of maximum intensity (FWHM) > 0.1ppm] or/and a signal-to-noise ratio below 5 were excluded from the analysis. Metabolite concentrations are expressed as ratios over Cr, as absolute values are more susceptible to partial volume effects and less reliable. Out of a total of 22 spectra obtained, only 14 spectra (WSC=6, CC=8) met the established quality criteria and were considered for further analysis. The high number of excluded low-quality spectra (n = 8) is related to the anatomical site of the hypothalamus, which is very difficult to locate and often leads to non-optimal equipment adjustment.

**Supplementary Methods 6.** **Immunohistochemistry (IHC) protocol**

After baking the slides at 60°C for 20 minutes to melt paraffin, slides were rehydrated using a standard rehydration curve (3x5 min Xylene, 100% Ethanol, 70% Ethanol, H_2_0). Endogenous peroxidase activity was quenched by exposure to 3% H_2_0_2_ for 30 minutes. Heat-mediated antigen retrieval was conducted in either citrate buffer or Tris-buffered saline (pH6) by usage of microwave (for 10m) or autoclave (15psi, 121°C for 60m) dependent on the antibody. After slides had cooled down, tissue sections were placed in Shandon Sequenza units and washed with PBS or TBS-T. Primary antibodies were applied for 1h at RT in antibody diluent (S2022 Dako REAL Antibody Diluent). Sections were washed 2x in PBS or TBS-T and secondary antibody (Dako REAL EnVision Detection System, Peroxidase/DAB, Rabbit/Mouse, HRP) was applied according to manufacturer’s instructions for 40 minutes. Sections were washed again in PBS or TBS-T (2x5min) and exposed to 3,3‐diaminobenzidine (DAB) EnVision+ kit (Dako, Glostrup, Denmark) for 5 min after which sections were washed 2x5min with PBS or TBS-T. Sections were counterstained using Harris Hematoxylin (Leica, 3801560E) for 60s. Subsequently, sections were washed in water and dehydrated (H_2_O, 70% Ethanol, 100% Ethanol, Xylene) after which they were kept in xylene (Genta Medical, XYL050) until slides were mounted using DPX (Fisher Scientific, 12658646).

Specific antigen retrieval methods, primary antibody concentrations, and incubation times were adjusted and standardized as shown in **Supplementary Table 2.** Positive controls indicated by the manufacturer were used for antibody optimization. A secondary antibody only control condition was included as negative control.

**Supplementary Methods 7**. **Regional Analysis**

1.1 Regional Analysis: Quantitative analysis

Neuronal counts were performed on tissue sections stained for H&E, with counting frames created at x400 magnification. Neuron cells were counted if they contained both a nucleus and a single, large nucleolus. Neuronal counts in each tissue section were averaged. Neuronal density was calculated by dividing the number of cells by the area of the counting frame.

Automated quantification of specific markers for neuroglia cells (Iba1- microglia, CD68- macrophages, and GFAP- astrocytes) was performed using Qupath Software and the algorithms described in **Supplementary Table 3**. Number of positively and negatively stained pixels were measured to generate a value for positivity (positivity = total number of positive pixels ÷ total area). A DAB‐stained image for each marker was used to threshold the algorithms ensuring all positively stained pixels were identified and counted. This quantification was performed exclusively on the gray matter of each brain region of interest. A section of control tissue, stained only with hematoxylin, was used to confirm the algorithm specificity.

1.2. Regional Analysis: Semi-quantitative analysis

The global measure of mTOR activity was assessed using semi‐quantitative expression analyses for each marker related to the mTOR pathway.

The semi‐quantitative analyses of Hamartin (TSC1), Tuberin (TSC2), mTOR, phospo-mTOR, ribosomal protein S6 kinase (p70S6K) and phospo-p70S6K markers staining were performed according to previously published methods ^S50^. Each marker was scored as absent (0), mild/some (1), moderate (2), or abundant (3) in each region of interest (scales showed in **Supplementary Figure 1-6**).

**Supplementary Methods 8. Neuroimaging statistical analysis**

Total GM, WM and CSF volumes (in milliliters) were calculated by the MATLAB get_totals script (http://www.cs.ucl.ac.uk/staff/g.ridgway/vbm/get_totals.m) implemented for SPM using the modulated segmented images for each subject. Between-group differences in total GM, CSF and WM volumes, total brain volume (TBV, calculated as GM + WM), and TIV (calculated as GM + WM + CSF) were tested using Unparied T-test.

Regarding VBM analyses, between-group statistical comparisons of mean GM and WM volumes were performed with the general linear model, based on random Gaussian field theory ^S51^. Firstly, we conducted exploratory between-group (WSC and CC) GM and WM volumes whole-brain comparisons; resulting statistics were thresholded at a Z value of ≥3.09 (corresponding to the two-tailed p≤0.001 level, uncorrected for multiple comparisons) and displayed as statistical parametric maps (SPMs) into standard anatomical space, with a minimum cluster size of 25 voxels. A measure of the total GM and WM volumes of each subject was entered as a confounder in an analysis of covariance model. Clusters of between-group GM and WM volumes difference were reported as significant with peak voxel surviving family-wise error (FWE) correction for multiple comparisons (p<0.05) over the entire brain. Secondly, we used the small volume correction (SVC) approach to conduct a hypothesis-driven investigation of the GM volumes where abnormalities have been identified in neuroimaging studies: caudate nucleus and putamen left; caudate nucleus and putamen right amygdala left; amygdala right; hypothalamus left; and hypothalamus, defined with basis on the Automated Anatomical Labeling (AAL) atlas. Thirdly, we used the small volume correction (SVC) approach to conduct a hypothesis-driven investigation of the WM volumes where abnormalities have been identified in neuroimaging studies: internal and external capsule, cingulum, fornix/stria terminalis, and corpus callosum, defined with basis on the JHU DTI-based WM atlases ^S52-54^. Findings of these hypothesis-driven, SVC-analyses were reported as significant if they survived FWE correction for multiple comparisons (pFWE≤0.05) over the specific brain region ^S51^.

For DTI analyses, voxel-based comparisons of FA values were performed using SPM12. FA values were standardized to the global mean using proportional scaling, thus controlling for interindividual differences in FA values. Firstly, we conducted exploratory between-group (WSC and CC) whole-brain comparisons; resulting statistics were thresholded at a Z value of≥3.09 (corresponding to the two-tailed p≤0.001 level, uncorrected for multiple comparisons) and displayed as statistical parametric maps (SPMs) into standard anatomical space, with a minimum cluster size of 25 voxels. Clusters of between-group of FA values difference were reported as significant with peak voxel surviving family-wise error (FWE) correction for multiple comparisons (p<0.05) over the entire brain. We used the small volume correction (SVC) approach to conduct a hypothesis-driven investigation of the WM volumes where abnormalities have been identified in neuroimaging studies: internal and external capsule, cingulum, fornix/stria terminalis, and corpus callosum, defined with basis on the JHU DTI-based WM atlases ^S52-54^. Findings of these hypothesis-driven, SVC-analyses were reported as significant if they survived FWE correction for multiple comparisons (pFWE≤0.05) over the specific brain region ^S51^.

For resting state fMRI analyses, we conducted seed-to-voxel analyses from a priori regions of interest (ROIs). For each ROI, whole-brain seed-to-voxel analyses were conducted to identify voxels with highly correlated time-courses. Functional connectivity maps showed the group comparison (CC cachexia > WSC controls) for a seed-to-voxel analysis using the parametric statistics (Gaussian Random Field theory) cluster threshold, two-tailed t-tests: p<0.05 cluster-size with a false discovery rate (FDR) corrected and voxel threshold: p<0.001 p-uncorrected. The ROIs for seed-to-voxel analysis were automatically defined using the standard brain atlas and networks of CONN toolbox. Three subjects were removed after pre-processing because there weren't enough valid scans (less than 3 min of total scan time) to guarantee the accuracy of the data. Results were corrected for multiple comparisons using false discovery rate (FDR) and significance level of p<0.05.

Data obtained using MRS, focused on the hypothalamus, was analyzed using Graphpad Prism 8.0. Comparisons between metabolites were conducted through Unpaired T-test. Significance was considered at p <0.05.

**Supplementary Table 1** Case Demographics of Post-Mortem Human Tissue Selected

PMI: post mortem interval. CERAD: The Consortium to Establish a Registry for Alzheimer’s disease. Braak: Neurofibrillary tangles staging.

**Supplementary Table 2** Optimized antibodies

| Target | Primary Antibody | Antibody Concentration | Clone | Antigen Retrieval | Incubation Settings | Positive Control |
| --- | --- | --- | --- | --- | --- | --- |
| Iba1 | Wako 019-19741 | 1:1000 in PBS | Polyclonal | Autoclave citrate | RT 1h |  |
| GFAP | Dako z0334 | 1:2500 | Polyclonal | MW citrate | RT 1h |  |
| CD68 | DakoCytomation M0876 | 1:50 | Clone PG-M1 | Autoclave citrate | RT 1h | Liver |
| TSC1 | Abcam ab40872 | 1:400 | Clone EP318Y | Autoclave tris EDTA | RT 1h | Cerebellum |
| TSC2 | Abcam ab32554 | 1:400 | Clone Y320 | Autoclave citrate | RT 1h | Cerebellum |
| mTOR | Cell signaling 2983 | 1:250 | Clone 7C10 | Autoclave citrate | RT 1h | Liver |
| pmTOR | Cell signaling 2976 | 1:100 | Clone 49F9 | Autoclave citrate | RT 1h | Breast carcinoma |
| p70S6K | Cell signaling 2708 | 1:100 | Clone 49D7 | Autoclave tris EDTA | RT 1h | Breast carcinoma |
| p-p70S6K | Invitrogen PA5-38307 | 1:200 | Polyclonal | Autoclave citrate | RT 1h | Breast carcinoma |

**Supplementary Table 3** Pixel counting Qupath algorithms

| **Target** | **Downsample factor** | **Gaussian sigma (μm)** | **Hematoxylin threshold (OD units)** | **DAB threshold (OD units)** |
| --- | --- | --- | --- | --- |
| Iba1 | 1.0 | 2 | 0.12 | 0.18 |
| GFAP | 1.0 | 2 | 0.15 | 0.2 |
| CD68 | 1.0 | 2 | 0.17 | 0.16 |

**Supplementary Figure 1** TSC1 semi-quantitative scale per region of interest

**Supplementary Figure 2** TSC2 semi-quantitative scale per region of interest

**Supplementary Figure 3** mTOR semi-quantitative scale per region of interest

**Supplementary Figure 4** phospho-mTOR semi-quantitative scale per region of interest

**Supplementary Figure 5** p70S6K semi-quantitative scale per region of interest

**Supplementary Figure 6** phospho-p70S6K semi-quantitative scale per region of interest

**Supplementary References**

S1. Baracos, V. E., Mazurak, V. C. & Bhullar, A. S. Cancer cachexia is defined by an ongoing loss of skeletal muscle mass. *Ann Palliat Med* **8**, 3–12 (2019).

S2. Blum, D. *et al.* Cancer cachexia: A systematic literature review of items and domains associated with involuntary weight loss in cancer. *Crit Rev Oncol Hematol* **80**, 114–144 (2011).

S3. Wyart, E. *et al.* Cachexia, a systemic disease beyond muscle atrophy. *International Journal of Molecular Sciences* vol. 21 1–18 Preprint at https://doi.org/10.3390/ijms21228592 (2020).

S4. Laviano, A. *et al.* Neural control of the anorexia-cachexia syndrome. 1000–1008 (2008) doi:10.1152/ajpendo.90252.2008.

S5. Molfino, A., Gioia, G., Fanelli, F. R. & Laviano, A. Contribution of Neuroinflammation to the Pathogenesis of Cancer Cachexia. *Mediators Inflamm* **2015**, (2015).

S6. Suzuki, H., Asakawa, A., Amitani, H., Nakamura, N. & Inui, A. Cancer cachexia—pathophysiology and management. *J Gastroenterol* **48**, 574–594 (2013).

S7. Ebadi, M. & Mazurak, V. C. Potential Biomarkers of Fat Loss as a Feature of Cancer Cachexia. *Mediators Inflamm* **2015**, (2015).

S8. Frank, M. G., Baratta, M. V., Sprunger, D. B., Watkins, L. R. & Maier, S. F. Microglia serve as a neuroimmune substrate for stress-induced potentiation of CNS pro-inflammatory cytokine responses. *Brain Behav Immun* **21**, 47–59 (2007).

S9. DiSabato, D., Quan, N. & Godbout, J. P. Neuroinflammation: The Devil is in the Details. *J Neurochem* **139**, 136–153 (2016).

S10. Laviano, A., Seelaender, M., Rianda, S., Silverio, R. & Rossi Fanelli, F. Neuroinflammation: a contributing factor to the pathogenesis of cancer cachexia. *Crit Rev Oncog* **17**, 247–51 (2012).

S11. Latacz, A., Russell, J. A., Ocloń, E., Zubel-Lojek, J. & Pierzchala-Koziec, K. mTOR Pathway - Novel Modulator of Astrocyte Activity. *Folia Biol (Praha)* **63**, 95–105 (2015).

S12. Henry, B. Loss of Tsc1 / Tsc2 activates mTOR and disrupts PI3K-Akt signaling through downregulation of PDGFR. **112**, 1223–1233 (2003).

S13. Dello Russo, C., Lisi, L., Tringali, G. & Navarra, P. Involvement of mTOR kinase in cytokine-dependent microglial activation and cell proliferation. *Biochem Pharmacol* **78**, 1242–1251 (2009).

S14. Kosillo, P. *et al.* Tsc1-mTOR signaling controls the structure and function of midbrain dopamine neurons. *bioRxiv* 376814 (2018) doi:10.1101/376814.

S15. Andersson, J. L. R., Jenkinson, M. & Smith, S. Non-linear optimisation FMRIB Technial Report TR07JA1. (2007).

S16. Whitfield-Gabrieli, S. & Nieto-Castanon, A. Conn: a functional connectivity toolbox for correlated and anticorrelated brain networks. *Brain Connect* **2**, 125–41 (2012).

S17. Provencher, S. W. Automatic quantitation of localized in vivo 1H spectra with LCModel. *NMR Biomed* **14**, 260–4 (2001).

S18. Gill, S. K., Ishak, M. & Rylett, R. J. Exposure of nuclear antigens in formalin-fixed, paraffin-embedded necropsy human spinal cord tissue: Detection of NeuN. *J Neurosci Methods* **148**, 26–35 (2005).

S19. Bankhead, P. *et al.* QuPath: Open source software for digital pathology image analysis. *Sci Rep* **7**, 16878 (2017).

S20. Takeuchi, H. *et al.* The associations of BMI with mean diffusivity of basal ganglia among young adults with mild obesity and without obesity. *Sci Rep* **10**, 12566 (2020).

S21. Bach, P. *et al.* Reliability of neural food cue-reactivity in participants with obesity undergoing bariatric surgery: a 26-week longitudinal fMRI study. *Eur Arch Psychiatry Clin Neurosci* **271**, 951–962 (2021).

S22. Steward, T., Menchon, J. M., Jiménez-Murcia, S., Soriano-Mas, C. & Fernandez-Aranda, F. Neural Network Alterations Across Eating Disorders: A Narrative Review of fMRI Studies. *Curr Neuropharmacol* **16**, 1150–1163 (2017).

S23. Azevedo, E. P., Ivan, V. J., Friedman, J. M. & Stern, S. A. Top-down control of appetite. *Biol Psychiatry* **91**, 869 (2022).

S24. Coppin, G. The anterior medial temporal lobes: Their role in food intake and body weight regulation. *Physiol Behav* **167**, 60–70 (2016).

S25. Giuliani, N. R., Merchant, J. S., Cosme, D. & Berkman, E. T. Neural predictors of eating behavior and dietary change. *Ann N Y Acad Sci* **1428**, 208–220 (2018).

S26. Kullmann, S. *et al.* Functional network connectivity underlying food processing: disturbed salience and visual processing in overweight and obese adults. *Cereb Cortex* **23**, 1247–1256 (2013).

S27. Rolls, E. T. The functions of the orbitofrontal cortex. *Brain Cogn* **55**, 11–29 (2004).

S28. Florio, T. M. *et al.* The Basal Ganglia: More than just a switching device. *CNS Neuroscience and Therapeutics* vol. 24 677–684 Preprint at https://doi.org/10.1111/cns.12987 (2018).

S29. Drummen, M. *et al.* Associations of Brain Reactivity to Food Cues with Weight Loss, Protein Intake and Dietary Restraint during the PREVIEW Intervention. *Nutrients* **10**, (2018).

S30. Rolls, E. T. Taste, olfactory, and food reward value processing in the brain. *Prog Neurobiol* **127–128**, 64–90 (2015).

S31. Lira, F. S. *et al.* Hypothalamic inflammation is reversed by endurance training in anorectic-cachectic rats. *Nutr Metab (Lond)* **8**, (2011).

S32. Lv, R. *et al.* Pathophysiological mechanisms and therapeutic approaches in obstructive sleep apnea syndrome. *Signal Transduct Target Ther* **8**, (2023).

S33. Liu, K. *et al.* Altered gray matter volume in children with newly diagnosed type 1 diabetes mellitus. *Pediatric Research 2022 93:5* **93**, 1342–1347 (2022).

S34. Kandel, E. R., Schwartz, J. H. ; & Jessell, T. M. *Principles of Neural Science* . (McGraw-Hill, New York, 2013).

S35. Molfino, A., Gioia, G., Fanelli, F. R. & Laviano, A. Contribution of Neuroinflammation to the Pathogenesis of Cancer Cachexia. *Mediators Inflamm* **2015**, 1–7 (2015).

S36. Yule, M. S., Brown, L. R., Skipworth, R. J. E. & Laird, B. J. A. Central neural mechanisms of cancer cachexia. *Curr Opin Support Palliat Care* (2024) doi:10.1097/SPC.0000000000000707.

S37. Liu, Y. J. *et al.* Peripheral T cells derived from Alzheimer’s disease patients overexpress CXCR2 contributing to its transendothelial migration, which is microglial TNF-α-dependent. *Neurobiol Aging* **31**, 175–188 (2010).

S38. Lai, J. *et al.* Fractional amplitude of low frequency fluctuation in drug-naïve first-episode patients with anorexia nervosa: A resting-state fMRI study. *Medicine (United States)* **99**, (2020).

S39. Ransohoff, R. M. How neuroinflammation contributes to neurodegeneration. *Science* vol. 353 777–783 Preprint at https://doi.org/10.1126/science.aag2590 (2016).

S40. Ebadi, M. & Mazurak, V. C. Potential Biomarkers of Fat Loss as a Feature of Cancer Cachexia. *Mediators Inflamm* **2015**, 1–8 (2015).

S41. Bachiller, S. *et al.* Microglia in Neurological Diseases: A Road Map to Brain-Disease Dependent-Inflammatory Response. *Front Cell Neurosci* **12**, 488 (2018).

S42. DiSabato, D., Quan, N. & Godbout, J. P. Neuroinflammation: The Devil is in the Details. *J Neurochem* **139**, 136–153 (2016).

S43. Molofsky, A. V *et al.* Astrocytes and disease: a neurodevelopmental perspective. *Genes Dev* **26**, 891–907 (2012).

S44. Ashburner, J. & Friston, K. J. Unified segmentation. Neuroimage 26, 839–851 (2005).

S45. Ashburner, J. A fast diffeomorphic image registration algorithm. Neuroimage 38, 95–113 (2007).

S46. Rueckert, D. Nonrigid registration using free-form deformations: Application to breast mr images. IEEE Trans Med Imaging 18, 712–721 (1999).

S47. Behzadi, Y., Restom, K., Liau, J. & Liu, T. T. A component based noise correction method (CompCor) for BOLD and perfusion based fMRI. Neuroimage 37, 90–101 (2007).

S48. Hallquist, M. N., Hwang, K. & Luna, B. The nuisance of nuisance regression: spectral misspecification in a common approach to resting-state fMRI preprocessing reintroduces noise and obscures functional connectivity. Neuroimage 82, 208–25 (2013).

S49. Kreis, R. Issues of spectral quality in clinical 1H-magnetic resonance spectroscopy and a gallery of artifacts. NMR Biomed 17, 361–81 (2004).

S50. Jenkins, D. R., Craner, M. J., Esiri, M. M. & DeLuca, G. C. Contribution of Fibrinogen to Inflammation and Neuronal Density in Human Traumatic Brain Injury. J Neurotrauma 35, 2259–2271 (2018).

S51 Friston, K. J. et al. Spatial registration and normalization of images. Hum Brain Mapp 3, 165–189 (1995).

S52. Hua, K. et al. Tract probability maps in stereotaxic spaces: analyses of white matter anatomy and tract-specific quantification. Neuroimage 39, 336–47 (2008).

S53. MRI Atlas of Human White Matter. AJNR Am J Neuroradiol 27, 1384 (2006).

S54. Wakana, S. et al. Reproducibility of quantitative tractography methods applied to cerebral white matter. Neuroimage 36, 630–44 (2007).
