# Supplementary material for: Cachexia Alters Central Nervous System Morphology and Functionality in Cancer Patients
Source: J Cachexia Sarcopenia Muscle. 2025 Feb 17;16(1):e13742. doi: 10.1002/jcsm.13742 (PMC11832348; doi:10.1002/jcsm.13742)
Supplement: Supplementary file 3 — Data S3. Supporting Information. [file JCSM-16-e13742-s003.pdf]

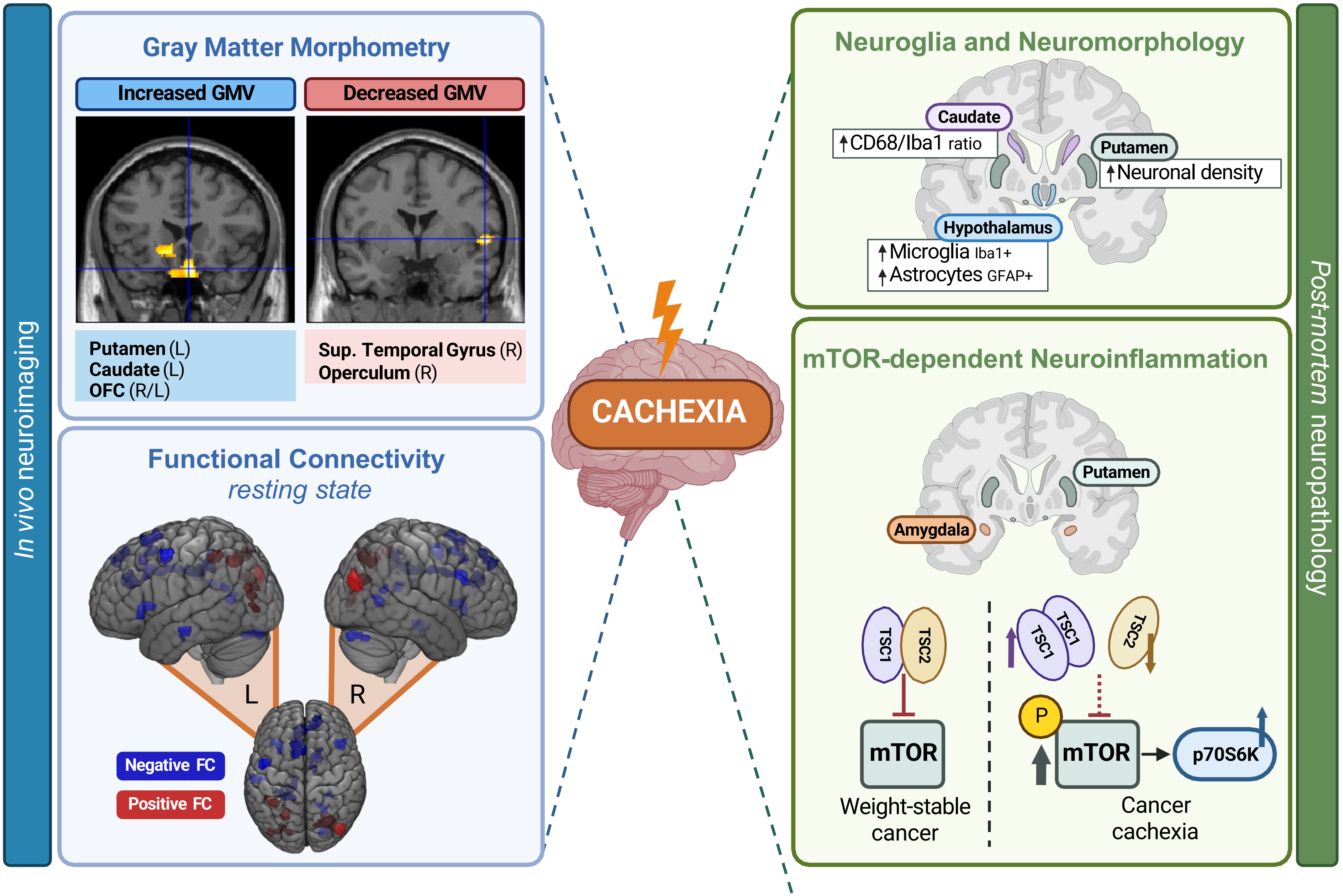

In vivo neuroimaging
Gray Matter Morphometry
Increased GMV
Decreased GMV
Putamen (L)
Caudate (L)
OFC (R/L)
Sup. Temporal Gyrus (R)
Operculum (R)
Functional Connectivity
resting state
L
R
Negative FC
Positive FC
CACHEXIA
Neuroglia and Neuromorphology
Caudate
↑CD68/Iba1 ratio
Putamen
↑Neuronal density
Hypothalamus
↑Microglia Iba1+
↑Astrocytes GFAP+
mTOR-dependent Neuroinflammation
Putamen
Amygdala
TSC1
TSC2
mTOR
Weight-stable cancer
TSC1
TSC1
TSC2
P
mTOR
p70S6K
Cancer cachexia
Post-mortem neuropathology
